# Interventions to Reduce Intra-Operative and Post-Operative Pain Associated with Routine Dental Procedures in Children: A Systematic Review and Meta-Analysis

**DOI:** 10.3390/dj12060163

**Published:** 2024-05-28

**Authors:** Mohammed A. Alzubaidi, Bernadette K. Drummond, Jianhua Wu, Adam Jones, Vishal R. Aggarwal

**Affiliations:** 1 Department of Preventive Dentistry, Faculty of Dentistry, Taif University, Taif 21944, Saudi Arabia; m.alzubaidi@tu.edu.sa; 2 Faculty of Medicine & Health, School of Dentistry, University of Leeds, Leeds LS2 9JT, UK; b.k.drummond@leeds.ac.uk (B.K.D.); j.h.wu@leeds.ac.uk (J.W.); a.jones4@leeds.ac.uk (A.J.); 3 Wolfson Institute of Population Health, Queen Mary University of London, London E1 4NS, UK

**Keywords:** dentistry, pain management, children, systematic review, meta-analysis

## Abstract

Objective: implementing appropriate pharmacological and non-pharmacological interventions to alleviate pain related to routine dental procedures in paediatric patients could enhance children’s ability to manage dental care. The aim of this review was to investigate the effectiveness of and provide recommendations for interventions that can be used to reduce intra-operative and post-operative pain associated with routine paediatric dental procedures. Methods: A systematic review of randomised controlled clinical trials (RCT) was conducted. Multiple electronic databases were systematically searched. The Cochrane risk-of-bias tool for RCTs was used to evaluate the quality of the included studies. A meta-analysis was performed to determine the effectiveness of the interventions using the Cohen’s d standardised mean differences (SMD) and 95% confidence intervals (CIs) for continuous outcomes. The GRADE tool was used to assess the certainty of evidence to make recommendations. Results: The review included forty-five RCTs comprising 3093 children. Thirty-seven RCTs were included in the meta-analysis, which showed the effectiveness of behavioural interventions (SMD = −0.50, 95% CI −0.83 to −0.18), mechanoreceptor and thermal receptor stimulation (SMD = −1.38, 95% CI −2.02 to −0.73) for intra-operative pain, and pre-emptive oral analgesics (SMD = −0.77, 95% CI −1.21 to −0.33) for reducing post-operative pain in children receiving routine dental care. Conclusion: The GRADE results for these interventions were strong recommendation (IB) for their use, based on moderate evidence and their benefits far outweighing the harm, and they can be delivered readily with minimal training to reduce the pain experience of paediatric patients.

## 1. Introduction

Dental caries in children is considered one of the major health problems that has a negative impact on a child’s and parent’s quality of life [1,2]. Globally, it affects 60–90% of schoolchildren [3]. Childhood dental caries still affects 23.4% of 5-year-old children in the UK [2]. Most paediatric dental procedures are carried out to treat dental caries in primary care and are conducted by general dental practitioners (GDPs) [3]. These procedures may comprise prevention, restorations, pulp treatments, extractions, and other dental care. These dental procedures may cause discomfort, pain, anxiety, and management difficulties which may require a pharmacological intervention [4,5]. A number of studies have investigated the influence of different dental procedures on childrens’ pain perception. For instance, Ghanei et al. (2018) reported that dental injection and extraction in children (aged 3 to 19 years) emerged as the most common causes of pain in paediatric dentistry, while drilling has been recognised as the second most common source of pain [6]. This is in agreement with a recent systematic review which showed extractions being the most painful dental procedure, followed by drilling [7]. Other studies considered dental injection as the procedure causing the highest pain [8,9,10]. Furthermore, in a recent study investigating the frequency and intensity of general and oral pain in Swedish children (aged 8 to 19 years), it was found that dental injection, drilling, or extraction were experienced as painful by half the young participants receiving invasive dental procedures [11]. Therefore, pain associated with a dental procedure might result in loss of co-operation and be a factor in exposing children to general anaesthesia (GA) if dental treatment can no longer be carried out under local anaesthesia [12]. Due to the drawbacks associated with GA in terms of risks, costs, and availability, it should be discouraged and only considered when other methods have failed or are not appropriate. 

The aim of this systematic review was to evaluate the effectiveness of and provide recommendations for interventions that can be used to reduce intra-operative and post-operative pain associated with routine paediatric dental procedures.

## 2. Material and Methods

This systematic review and meta-analysis were undertaken following the Preferred Reporting Items for Systematic Reviews and Meta-Analyses (PRISMA) statement [13,14]. The protocol was registered and published in the International Prospective Register of Systematic Reviews (PROSPERO; registration number: CRD42020177771).

### 2.1. Inclusion Criteria

Studies comprising single-, double-, or triple-blinded randomised controlled clinical trials that investigated interventions to reduce pain related to routine dental procedures in children were included. The research question was ‘what are the interventions that can reduce intra-operative and post-operative pain associated with routine dental procedures in children?’. The PICO strategy used for this review was as follows: Population (P): children and adolescents regardless of medical and behavioural problems receiving routine dental treatment with or without local anaesthesia (LA); intervention (I) and comparison (C): a consensus panel consisting of the author (MAA), a consultant in paediatric dentistry (BKD), an oral surgeon with methodological expertise in systematic reviews (AJ), and a primary care dentist with a special interest in pain (VRA) met after the results of the preliminary search and grouped the interventions according to method of local anaesthetic (LA) delivery (computer driven, intraosseous, use of topical anaesthesia versus conventional LA), pharmacological agents, behavioural interventions, and those that interrupted pain pathways through mechano–receptor stimulation; and outcome (O): the primary outcomes that were considered for this review were intra-operative and post-operative pain measured using a validated pain scale. Anxiety measured using a validated anxiety scale was also considered as a secondary outcome.

### 2.2. Exclusion Criteria

Studies involving dental treatment under general anaesthesia or sedation were excluded. 

### 2.3. Search Strategy

In this review, electronic databases were meticulously queried using comprehensive search strategies for identifying studies that met the eligibility criteria. The author (MAA) formulated the search strategies under the guidance of an expert librarian at the University of Leeds (Supplementary File S1). The approach to searching involved a mix of controlled vocabulary and free text terms to ensure the identification of eligible studies, irrespective of the language or publication date. The following electronic databases were searched up to the 11th of March 2022: Cochrane Library (Wiley), MEDLINE via OVID, EMBASE via OVID, PsycINFO via OVID, WHO International Clinical Trials Registry Platform, Clinical Trials.gov, Web of Science, and PubMed. Additionally, the bibliographies of included studies were screened to uncover further studies that qualified for inclusion. 

### 2.4. Data Extraction

All references were transferred to EndNote version X9 [15] and subsequently managed through Covidence systematic review software [16], where duplicate records were detected and excluded. The two review authors (MAA and AJ) independently assessed the titles and abstracts of relevant articles. Any disagreement was resolved by discussion and consensus with the third reviewer (VRA).

Studies that met the inclusion criteria were accepted regardless of their methodological quality. Study authors were approached for additional information in instances of missing data or inconsistent reporting. Two reviewers (MAA and AJ) independently extracted the required information using Covidence systematic review software. Extracted characteristics of the studies included name of the first author, journal of publication, year of publication, country, sitting, study design, population and participant characteristics, sample size, dental procedure, intervention, type of outcome, and methods of measurement.

### 2.5. Quality Assessment

The quality assessment of the selected studies was conducted independently by the same reviewers (MAA and AJ) following the guidance of the *Cochrane Handbook for Systematic Reviews of Interventions* [17]. The following seven domains were assessed for quality of included studies: sequence generation, allocation concealment, blinding of participants and personnel, blinding of outcome assessors, incomplete outcome data, selective outcome reporting, and any other bias relevant to the study [17]. A study was considered as at low risk of bias when all seven domains were judged as low risk, unclear risk of bias when any domain was judged as unclear risk, and high risk of bias when any domain was judged as high risk. Any disagreement was resolved by discussion and consensus with the third reviewer (VRA). 

### 2.6. Data Analysis

All extracted data were exported and managed in an Excel spreadsheet (Microsoft Inc., Washington, DC, USA) [18]. The data extraction form was subsequently refined to facilitate the analytical process.

Meta-analysis was conducted when an adequate number of studies reported similar outcomes, aiming to enhance statistical power through pooling the results of these studies. The Cohen’s d standardised mean differences (SMD) 95% confidence intervals (CIs) were considered for continuous outcomes that were measured with different pain or anxiety scales to estimate the efficacy of interventions used to relieve pain and anxiety associated with routine dental treatment in children. Data collection for the meta-analysis included the following information: name of the first author, dental procedures included, intervention used, pain outcome (mean, standard deviation (SD), number of participants (N), and standard of error (SE)), and anxiety outcome (mean, SD, N, and SE).

Stata 16 statistical software (StataCorp LLC, College Station, TX, USA) was used to undertake the meta-analysis and generate forest plots using a random-effects model [19]. A random-effects model was used to take into account heterogeneity between studies. 

Clinical heterogeneity among the included studies was accounted for by inclusion criteria for studies, participants, components of the interventions, and outcome measures. Statistical heterogeneity was evaluated using I^2^ statistics, where values equal to or exceeding 50% indicated substantial heterogeneity. A *p*-value 0.05 or lower was deemed to denote statistical significance. 

### 2.7. Strength/Certainty of Evidence 

Based on the *Cochrane Handbook for Systematic Reviews of Interventions*, the Grading of Recommendations Assessment, Development and Evaluation (GRADE) approach was applied to assess the certainty of the effect sizes of each intervention using the programme GRADEpro GDT [17]. The certainty of the evidence was downgraded from a high-quality level of evidence by one or more levels when there were limitations in the risk of bias, consistency of the results, and/or precision of the pooled estimate. Depending on the number of limitations, the strength or certainty of evidence was then graded as either high, moderate, low, or very low.

The GRADE recommendations, strong (I), moderate (IIa), weak (IIb), or recommended against (III), were based on the risks/benefits of the interventions and were collated according to strength of evidence as follows:

A: high level of evidence; consistent evidence.

B: moderate/low level of evidence; evidence with few important limitations. 

C: very low level of evidence; evidence with serious flaws.

## 3. Results

### 3.1. Study Selection

**Figure 1:**
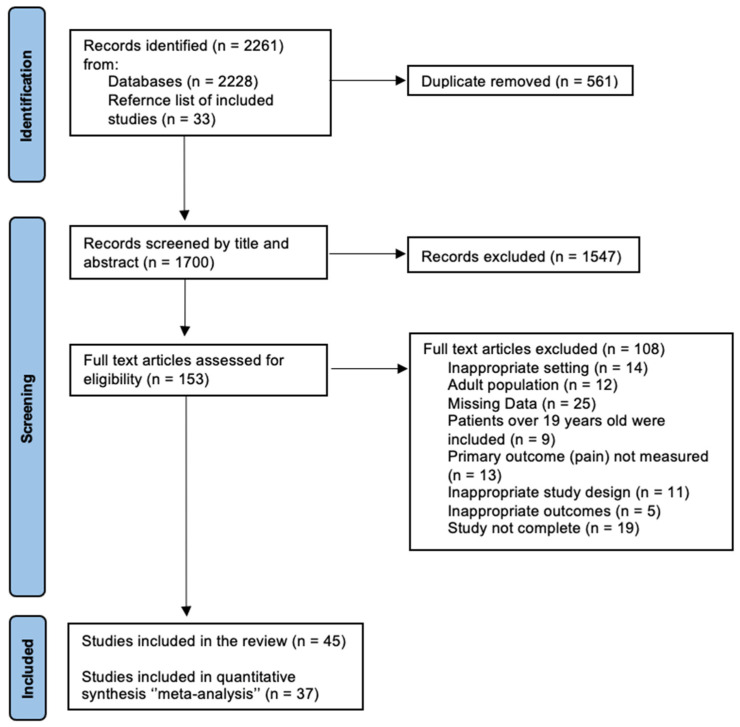
PRISMA flowchart of the study selection process.

A total of 2261 studies were identified after the electronic and manual search, while 1700 remained after excluding duplicates. Following the title and abstract screening, 153 articles were selected for full-text review and examined against the eligibility criteria in detail, and 1547 studies were excluded. Twenty-five studies had missing data; although the authors were contacted, these data were not provided. Fourteen studies had an inappropriate setting. Moreover, twelve studies involved an adult population, and nine studies included patients over 19 years of age. Five studies had inappropriate outcomes, and thirteen studies did not measure pain. Additionally, eleven studies had an inappropriate study design. Finally, nineteen studies were excluded because they were not complete. Consequently, forty-five studies were identified and included in the review, and thirty-seven studies were eligible for the meta-analysis (Figure 1). 

### 3.2. Study Characteristics

The characteristics of the included studies are summarised in Supplementary File S2. Twenty-five included studies adopted parallel group randomised controlled trials [20,21,22,23,24,25,26,27,28,29,30,31,32,33,34,35,36,37,38,39,40,41,42,43,44], whereas nineteen studies were crossover group randomised controlled trials [45,46,47,48,49,50,51,52,53,54,55,56,57,58,59,60,61,62,63] and one study used both parallel group and split-mouth randomised controlled trials [64]. 

The sample size ranged from 25 to 160, and each study had a different sample size, with a total number of 3093 children who had different interventions to relieve pain and/or anxiety associated with dental procedures.

Nine studies were carried out in India, five studies in Brazil, five studies in Saudi Arabia, four studies in the USA, four studies in Syria, four studies in Turkey, three studies in Iran, two studies in Egypt, two studies in the Netherlands, two studies in France, two studies in Mexico, one study in Italy, one study in Israel, and one study in Thailand.

Studies included children aged from three to sixteen years, and a different age range of children was considered in each study. One study did not report the age range of its sample but only included children with a maximum age of 16 years [21]. 

All studies reported on the primary outcomes of intra-operative and/or post-operative pain associated with paediatric dental procedures, while only nine studies assessed anxiety levels before and after the dental procedures [23,24,25,36,38,40,41,43,52]. Most of the included studies used self-report measures of pain scales which were dependent on the patient’s self-reporting. Also, different anxiety scales were considered to measure anxiety levels. The different methods used by authors to assess pain and anxiety are summarised in Supplementary File S2 (the characteristics of included studies).

### 3.3. Quality Assessment for Risk of Bias 

**Figure 2:**
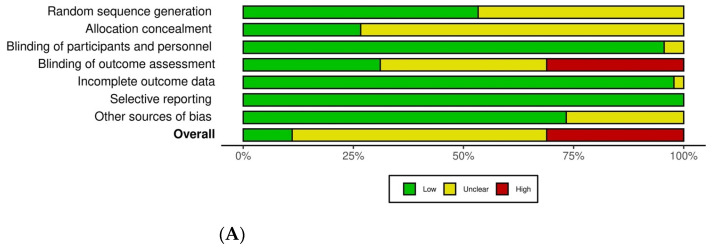

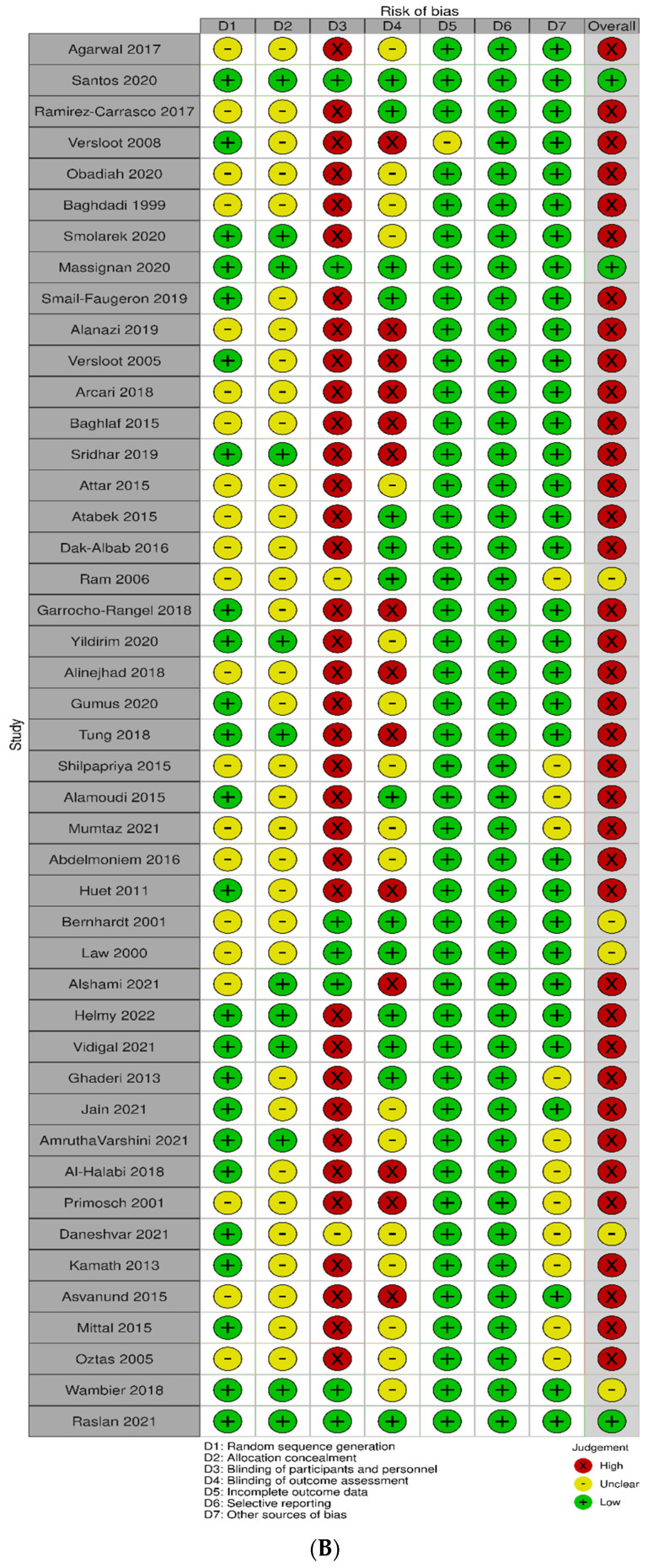
(**A**) Overall risk of bias. (**B**) Risk of bias for individual studies [20,21,22,23,24,25,26,27,28,29,30,31,32,33,34,35,36,37,38,39,40,41,42,43,44,45,46,47,48,49,50,51,52,53,54,55,56,57,58,59,60,61,62,63,64].

Figure 2 illustrates the overall risk of bias and the individual plots for each study related to sequence generation, allocation concealment, blinding (participants and outcome assessment), incomplete outcome reporting, and selection bias. Other sources of bias included not reporting on baseline participant characteristics [20,26,27,29,33,46,47,49,53,61,62] and one study did not report which LA agent was given by infiltration or mandibular block injections [48]. The details of the quality assessment of the included studies are reported in Supplementary File S2 (the characteristics of included studies).

The overall risk of bias for three studies was rated as low because all domains of the quality assessment for these studies were considered to be at low risk of bias [37,39,42]. Thirty-seven studies were judged to be at high risk of bias for at least one domain. The remaining five studies were rated as unclear risk of bias (Figure 2).

### 3.4. Meta-Analyses

#### 3.4.1. Local Anaesthesia

**Figure 3:**
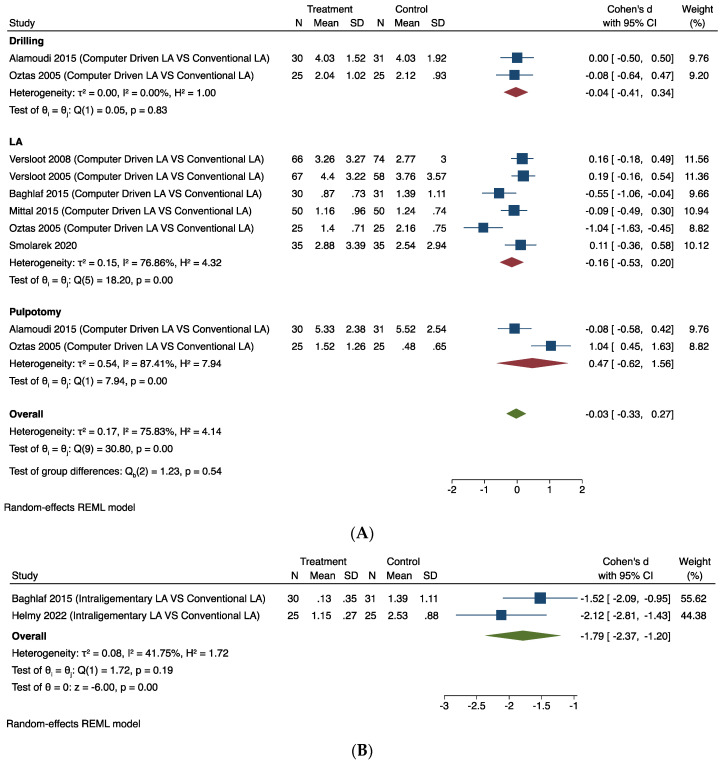

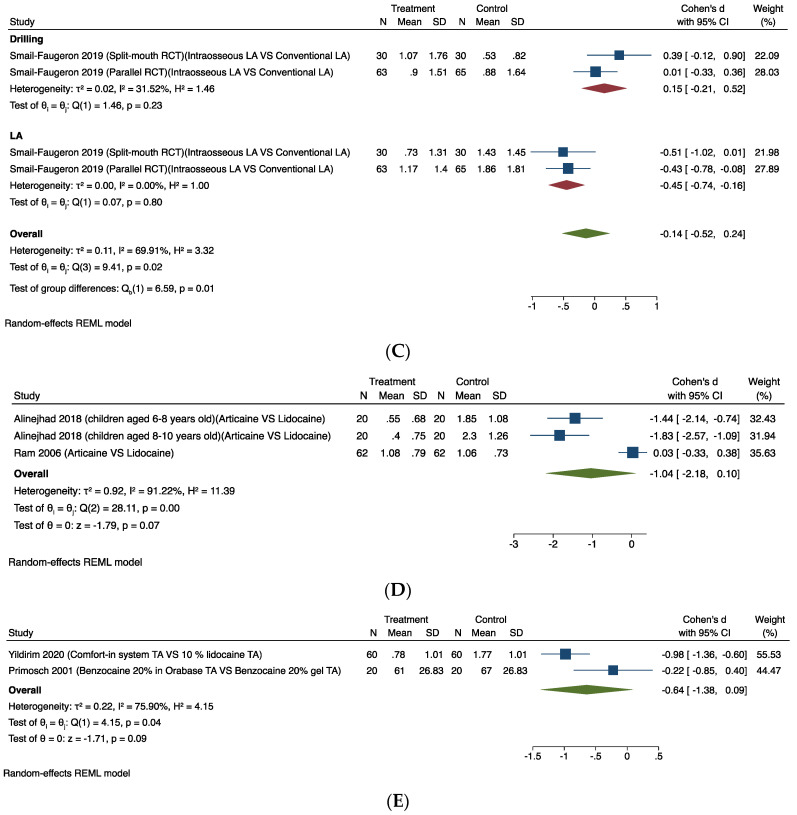
Random-effects meta-analysis evaluation of local anaesthesia for comparisons: (**A**) Computer-driven LA versus conventional LA. (**B**) Intra-ligamentary LA versus conventional LA. (**C**) Intraosseous LA versus conventional LA. (**D**) LA agent (articaine versus lidocaine). (**E**) Topical anaesthesia [23,24,27,28,29,40,47].

The forest plots for pain following local anaesthesia are shown in Figure 3.

##### Computer-Driven LA Versus Conventional LA

Seven studies provided comparable data for this outcome [23,24,27,28,29,40,47]. Figure 3A shows the results of the meta-analysis. Pooling the results of these studies using a random-effects model and the Cohen’s d standardised mean difference (SMD) showed that the use of computer-driven LA (infiltration/IANB) was no better than conventional LA in relieving intra-operative pain associated with dental procedures (SMD −0.03, 95% CI −0.33 to 0.27). The substantial heterogeneity (I^2^ = 75.83%) in this estimate is likely to be due to the different tools used to measure the outcomes and the ways of delivering LA via infiltration/IANB in each trial. 

##### Intraosseous/Intra-Ligamentary LA Versus Conventional LA

Two studies provided comparable data for intra-ligamentary LA versus conventional LA (IANB) outcomes [28,44]. Figure 3B shows the results of the meta-analysis. Pooling the results of these studies using a random-effects model and the Cohen’s d standardised mean difference (SMD) showed that using intra-ligamentary LA via the Wand was less painful than conventional LA (SMD −1.79, 95% CI −2.37 to −1.20). The heterogeneity is low (I^2^ = 41.75%) in this estimate. 

One trial using two study designs (parallel group and split-mouth RCTs) assessed pain experience in paediatric patients with intra-osseous LA using QuickSleeper^TM^ compared with conventional LA [64]. As there were two study designs in this trial, it was not possible to provide comparable data for this outcome. 

Figure 3C shows the results of the meta-analysis. Pooling the results of these studies using a random-effects model and the Cohen’s d standardised mean difference (SMD) showed that the use of intra-osseous LA did not significantly relieve intra-operative pain associated with dental procedures (LA and drilling) (SMD −0.14, 95% CI −0.52 to 0.24). The substantial heterogeneity (I^2^ = 69.91%) in this estimate is likely to be due to the different study designs used in this trial. 

##### LA Agent (Articaine Versus Lidocaine)

Two studies provided comparable data for this outcome [48,55]. Figure 3D shows the results of the meta-analysis. Pooling the results of these studies using a random-effects model and the Cohen’s d standardised mean difference (SMD) revealed that the use of articaine infiltration LA was not shown to significantly relieve intra-operative pain associated with LA (SMD −1.04, 95% CI −2.18 to 0.10). The considerable heterogeneity (I^2^ = 91.22%) in this estimate is likely to be due to the different tools used to measure the outcomes and the ways of delivering LA in each trial. 

##### Topical Anaesthesia

Two studies provided comparable data for this outcome [46,60]. Figure 3E shows the results of the meta-analysis. Pooling the results of these studies using a random-effects model and the Cohen’s d standardised mean difference (SMD) showed that there was no difference between different types of topical anaesthesia in relieving pain associated with LA (SMD −0.64, 95% CI −1.38 to 0.09). The substantial heterogeneity (I^2^ = 75.90%) in this estimate is likely to be because the studies considered different tools used to measure the outcome, different types of topical anaesthesia used, and the ways of delivering LA via infiltration/IANB in each trial.

#### 3.4.2. Intra-Operative Pain Outcome

**Figure 4:**
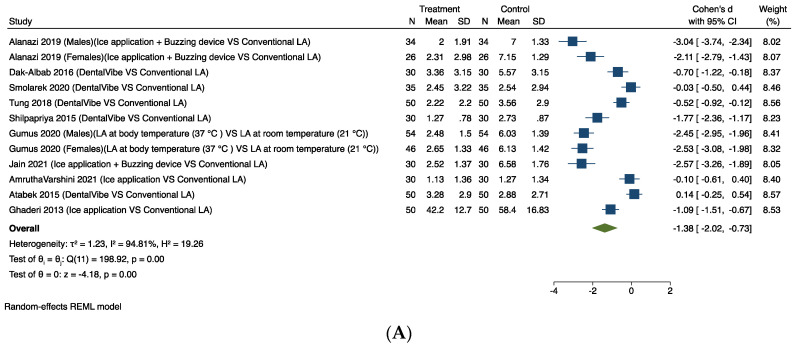

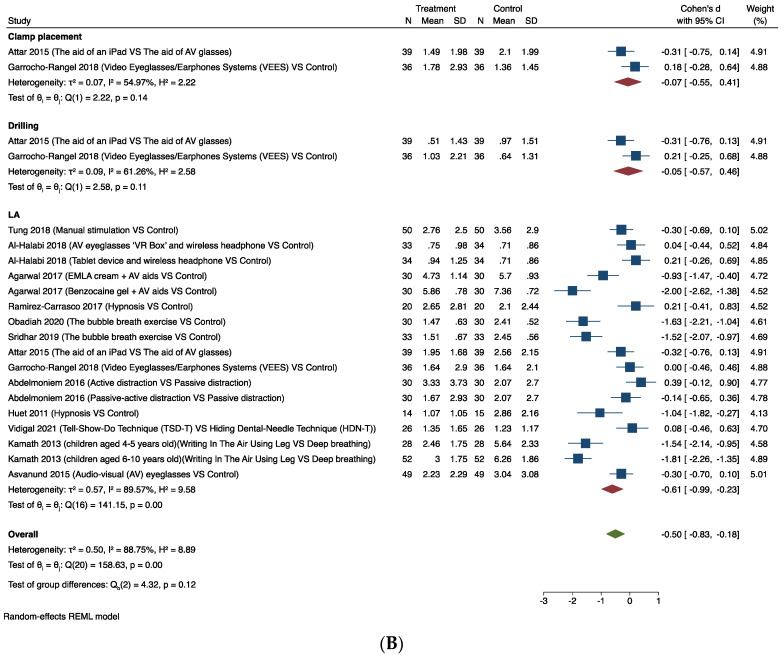
Random-effects meta-analysis evaluation of intra-operative pain for comparison: (**A**) Mechanoreceptor and thermal receptor stimulation. (**B**) Behavioural interventions [35,40,49,50,53,54,58,59,61,63].

The forest plots for the meta-analysis for intra-operative pain as an outcome are shown in Figure 4. The following interventions impacted on positively on intra-operative pain.

#### 3.4.3. Mechanoreceptor and Thermal Receptor Stimulation

Ten studies provided comparable data for this outcome [35,40,49,50,53,54,58,59,61,63]. Figure 4A shows the results of the meta-analysis. Pooling the results of these studies using a random-effects model and the Cohen’s d standardised mean difference (SMD) showed a significant difference in favour of using mechanoreceptor and thermal receptor stimulation to relieve intra-operative pain associated with LA (SMD −1.38, 95% CI −2.02 to −0.73). The considerable heterogeneity (I^2^ = 94.81%) in this estimate is likely to be due to the studies having considered different tools to measure the outcomes and the different mechanoreceptor and thermal receptor stimulation used in each trial. 

#### 3.4.4. Behavioural Interventions

Thirteen studies provided comparable data for this outcome [25,26,30,31,32,33,35,36,38,43,51,52,56]. Figure 4B shows the results of the meta-analysis. Pooling the results of these studies using a random-effects model and the Cohen’s d standardised mean difference (SMD) showed that the use of different behavioural interventions significantly relieved intra-operative pain associated with dental procedures (clamp placement, LA, and drilling) (SMD −0.50, 95% CI −0.83 to −0.18). The considerable heterogeneity (I^2^ = 88.75%) in this estimate is likely to be due to the different tools used to measure the outcomes and the different behavioural interventions used in each trial. 

#### 3.4.5. Post-Operative Pain Outcome

**Figure 5:**
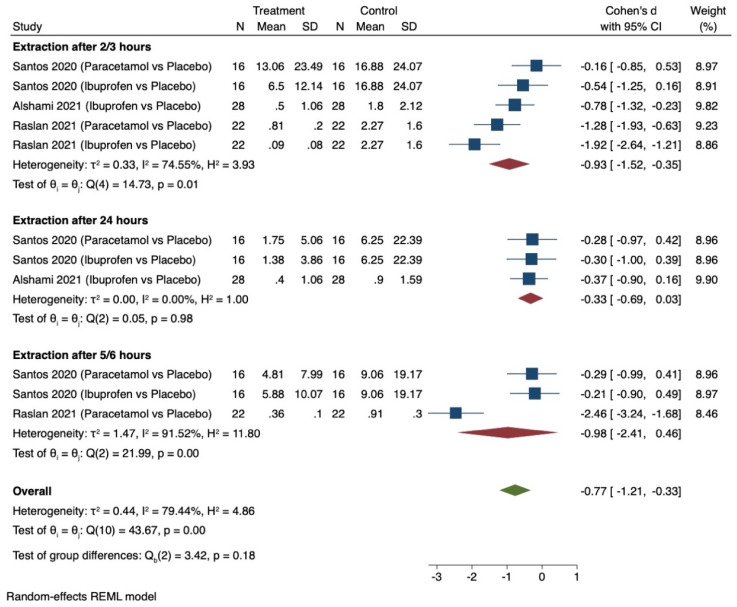
Random-effects meta-analysis evaluation of post-operative pain for comparison of pharmacological interventions (oral analgesics versus placebo) [36,38,43].

Only three studies provided comparable data for this outcome [39,41,42] and all related to pharmacological interventions. Figure 5 shows the results of the meta-analysis. Pooling the results of these studies using a random-effects model and the Cohen’s d standardised mean difference (SMD) showed that the use of pre-emptive analgesics significantly relieved post-operative pain associated with extraction between intervention and control groups (SMD −0.77, 95% CI −1.21 to −0.33). The considerable heterogeneity (I^2^ = 79.44%) in this estimate is likely to be due to the studies having considered different tools to measure the outcomes and the different pre-emptive analgesics used at different times in each trial. 

#### 3.4.6. Anxiety Outcome

Three studies provided comparable data for this outcome [36,38,43] and all related to behavioural interventions. Pooling the results of these studies using a random-effects model and the Cohen’s d standardised mean difference (SMD) showed no significant difference in reducing anxiety associated with dental procedures between intervention and control groups (SMD −0.17, 95% CI −0.45 to 0.11). The heterogeneity was low (I^2^ = 33.42%) in this estimate.

### 3.5. Certainty of Evidence and GRADE Recommendations

The certainty of the evidence was assessed using the GRADE criteria and considered to be moderate/low for interventions measuring intra-operative and post-operative pain and anxiety outcomes (Supplementary File S3). The main drivers for downgrading the certainty of evidence were the high risk of bias detected in some studies and the presence of imprecision. The GRADE recommendations were strong (IB) based on the moderate evidence and positive benefit outweighing the low risk/burden of mechanoreceptor and thermal receptor stimulation, behavioural interventions, and pre-emptive oral analgesics for reducing pain associated with routine dental care in children (Table 1).

## 4. Discussion

### 4.1. Summary of Main Findings

The findings of this systematic review support strong recommendations for the use of mechanoreceptor and thermal receptor stimulation to reduce intra-operative pain associated with local anaesthesia and the use of behavioural interventions to reduce intra-operative pain associated with local anaesthesia and drilling. For post-operative pain, there was evidence to support the use of pre-emptive analgesics for extractions. Evidence was inconclusive for the use of computer-driven LA devices in comparison to conventional injection of LA to reduce pain. This was due to the limited number of studies, and further high-quality trials for use of these devices are needed. 

### 4.2. Strengths and Limitations

We followed the PRISMA and Cochrane guidance to ensure rigour in the review process. We had a large sample with data assembled from forty-five studies comprising 3093 children who received routine dental care. Thirty-seven studies were included in the meta-analysis and provided nine comparisons, of which seven were on the outcome of intra-operative pain, one was on post-operative pain outcome, and one was on the anxiety outcome.

Some limitations are noted. More than 50% of studies were assessed as being at unclear risk of bias, mostly arising from issues related to sequence generation and concealment of allocation that might increase the risk of selection bias. The remaining studies were rated at high risk of bias due to unblinding of outcome assessors, which might increase the risk of detection bias, with the exception of five studies that were judged to be at low risk of bias. Due to the nature of the intervention, blinding of operators and/or participants was not possible in the majority of studies and we reflected this in the GRADE scores. Some studies were excluded because they had missing data which we could not obtain despite contact with the authors. 

### 4.3. Comparison with Previous Literature and Future Research

The findings of our review strengthen and support those of previous studies on the use of pre-emptive oral analgesics (paracetamol/ibuprofen) for relieving pain in children undergoing routine dental treatment, which found reduction in post-op pain using pre-operative analgesics before orthodontic separator placement [65]. Our review supports these findings in addition to supporting the use of pre-emptive analgesics 2–3 h after extraction of primary molars.

With regard to computer-driven versus conventional LA, our review found no beneficial effects of the Wand system when used as infiltration/IANB for different dental procedures. It did, however, significantly relieve intra-operative pain associated with LA for intra-ligamentary injections. These findings were in agreement with one other similar systematic review and meta-analysis conducted by Smolarek et al. (2020) who combined the Wand system and QuickSleeper^TM^ regardless of the injection site into a single meta-analysis and concluded that there were no differences in pain perception and disruptive behaviour between computerised and conventional dental local anaesthesia, but the quality of evidence was low [66].

Due to disparity in findings and the limited number of studies (only two studies were included in that meta-analysis) further research in the form of high-quality randomised controlled trials is therefore needed to support the use of computer-driven LA devices and to compare different types of LA agents. 

Several studies have investigated the effects of mechanoreceptor and thermal receptor stimulation on pain perception during LA injections. The working principle of these interventions can be explained by the gate control theory of pain [67]. The included studies demonstrated different mechanoreceptor and thermal receptor stimulation interventions such as vibrating devices including a buzzing device, DentalVibe, different LA temperatures, and ice application. The results of our review showed that pain perception was significantly relieved during LA injections when different types of mechanoreceptor and thermal receptor stimulation were applied and strongly supports the use of these interventions.

A considerable amount of literature has been published on the effectiveness of non-pharmacological (behavioural) interventions in reducing pain and anxiety in children receiving dental care [68]. The current review looked at different behavioural interventions such as TSD, hypnosis, bubble breath exercise, and distraction. The findings of this review strongly recommend that the use of behavioural interventions can significantly help reduce pain perception during LA injections. This finding seems to be consistent with another similar systematic review conducted by Goettems et al. (2017) who investigated the effectiveness of non-pharmacologic interventions on behaviour, anxiety, and pain perception in children undergoing dental treatment [69]. 

## 5. Conclusions

The findings of this systematic review support strong recommendations for the use of mechanoreceptor and thermal receptor stimulation to reduce intra-operative pain associated with local anaesthesia and the use of behavioural interventions to reduce intra-operative pain associated with local anaesthesia and drilling. This review also supports the use of pre-emptive analgesia to reduce post-operative pain for dental extractions and orthodontic separator placement. Further high-quality trials are needed to explore the effectiveness of computer-driven LA devices in reducing pain during LA injections, for which the review findings were inconclusive. The strong recommendations for use of behavioural interventions and mechanoreceptor and thermal receptor stimulation are based on the benefits of pain relief far outweighing the harms or burdens of these interventions. In addition, these interventions can be readily delivered with minimal training and in any dental setting, particularly primary care where the patient initially presents for treatment. Reduction of pain is likely to positively impact the patient experience and maintain co-operation, thus preventing referral to already stretched secondary care services for sedation or general anaesthesia for which children may have to wait more than 12 months.

## Figures and Tables

**Table 1:** The GRADE recommendations.

Intervention	Effect Size and Conclusions	GRADE Recommendation
Mechanoreceptor and thermal receptor stimulation (intra-operative pain outcome)	SMD 1.38, SD lower (2.02 lower to 0.73 lower) Moderate level of evidence	IB, strong recommendation based on the moderate level of evidence
Behavioural interventions (intra-operative pain outcome)	SMD 0.5, SD lower (0.83 lower to 0.18 lower) Moderate level of evidence	IB, strong recommendation based on the moderate level of evidence
Pharmacological interventions: oral analgesics (post-operative pain outcome)	SMD 0.77, SD lower (1.21 lower to 0.33 lower) Moderate level of evidence	IB, strong recommendation based on the moderate level of evidence

## Supplementary Materials

The following supporting information can be downloaded at: https://www.mdpi.com/article/10.3390/dj12060163/s1, S1: search strategy; S2: Characteristics of included studies; S3: GRADE assessment.
