# Supplementary material for: Interventions to Reduce Intra-Operative and Post-Operative Pain Associated with Routine Dental Procedures in Children: A Systematic Review and Meta-Analysis
Source: Dent J (Basel). 2024 May 28;12(6):163. doi: 10.3390/dj12060163 (PMC11202832; doi:10.3390/dj12060163)
Supplement: Supplementary file 1 [file dentistry-12-00163-s001.zip › Supplementary File S1.pdf]

Supplementary file S1: Search Strategy

MEDLINE via OVID
1. exp Dentistry/
2. (dental* or dentist*).ti,ab.
3. (oral adj5 surg*).ti,ab.
4. (orthodontic* or pulpotom* or pulpect* or endodont* or "pulp cap*").mp.
5. ((dental or tooth or teeth) adj5 (fill* or restor* or extract* or remov* or "cavity prep*" or caries or carious or decay*)).mp.
6. (root canal and (therap* or treat*)).mp.
7. child/
8. Infant/
9. adolescent/
10. Pediatrics/
11. Dental care for children/
12. (child* or adolescent* or kid or kids or youth* or youngster* or minor or minors or teen* or preteen* or pre-teen* or juvenile* or "young adult*" or "young person" or "young people" or p?ediatric* or student* or pupil or pupils or boy or boys or girl or girls or under 18* or under eighteen* or underage).ti,ab,kw.
13. Pain, Postoperative/
14. (postoperative adj4 pain*).ti,ab,kw.
15. (post-operative adj4 pain*).ti,ab,kw.
16. post-operative-pain*.ti,ab,kw.
17. (post* adj4 pain*).ti,ab,kw.
18. (postoperative adj4 analgesi*).ti,ab,kw.
19. (post-operative adj4 analgesi*).ti,ab,kw.
20. post-operative analgesi*.ti,ab,kw.
21. (post-surgical adj4 pain*).ti,ab,kw.
22. (post surgical adj4 pain*).ti,ab,kw.
23. (pain* adj4 after surg*).ti,ab,kw.
24. (pain* adj4 after operat*).ti,ab,kw.
25. (pain* adj4 follow* operat*).ti,ab,kw.
26. (pain* adj4 follow* surg*).ti,ab,kw.
27. (pain adj1 operati*).ti,ab,kw.
28. (intra adj procedur* adj pain).ti,ab,kw.
29. (intraoperative adj4 pain*).ti,ab,kw.
30. (intra-operative adj4 pain*).ti,ab,kw.
31. intra-operative-pain*.ti,ab,kw.
32. (intraoperative adj4 analgesi*).ti,ab,kw.
33. (intra-operative adj4 analgesi*).ti,ab,kw.
34. intra-operative analgesi*.ti,ab,kw.
35. (intra-surgical adj4 pain*).ti,ab,kw.
36. (intrasurgical adj4 pain*).ti,ab,kw.
37. (pain* adj4 after surg*).ti,ab,kw.
38. (pain* adj4 after operat*).ti,ab,kw.
39. (pain* adj4 follow* operat*).ti,ab,kw.
40. (pain* adj4 follow* surg*).ti,ab,kw.
41. (surg* adj1 pain).ti,ab,kw.
42. (("post surg*" or post-surg*) and (pain* or discomfort)).ti,ab,kw.
43. (analgesi* adj4 surg*).ti,ab,kw.

44. (analgesi* adj4 operat*).ti,ab,kw.
45. (sore* or hurt* or ache* or aching or discomfort* or uncomfort* or tender* or throb*).mp. [mp=title, abstract, original title, name of substance word, subject heading word, floating sub-heading word, keyword heading word, organism supplementary concept word, protocol supplementary concept word, rare disease supplementary concept word, unique identifier, synonyms]
46. An?esthetics, Local/
47. An?esthesia, Local/
48. (local adj5 (anesthetic* or anaesthetic* or anesthesia or anaesthesia)).mp.
49. Lidocaine/
50. (lidocaine or lignocaine or xylocaine).mp.
51. Carticaine/
52. (carticain* or articain*).mp.
53. Prilocaine/
54. (prilocain* or citanest* or propitocain* or xylonest).mp.
55. Bupivacaine/
56. (bupivacain* or buvacaina or carbostesin or dolanaest or marcain* or sensorcain* or svedocain*).mp.
57. Analgesi*.ti,ab,kw.
58. (anti-inflammatory or nitrous oxide sedation).mp. [mp=title, abstract, original title, name of substance word, subject heading word, floating sub-heading word, keyword heading word, organism supplementary concept word, protocol supplementary concept word, rare disease supplementary concept word, unique identifier, synonyms]
59. (nonsteroidal anti-inflammatory agent* or anti inflammatory agent* or nonsteroidal antiinflammatory agent* or non steroidal antiinflammatory agent* or Nonsteroidal analgesic* or anti-inflammator* or aspirin-like agent* or NSAID*).ti,ab,kw.
60. (ibuprofen or brufen).ti,ab,kw.
61. (acetaminophen or paracetamol).ti,ab,kw.
62. pain management/
63. (pain adj3 management).tw.
64. psychotherapy/
65. psychotherap*.tw.
66. behavior therapy/
67. (behavi?r adj3 therap*).tw.
68. cognitive therapy/
69. (cognitive adj3 therap*).tw.
70. (relax* adj3 (technique* or therap*)).tw.
71. exp mind body therapies/
72. guided imagery.tw.
73. "imagery (Psychotherapy)"/
74. mindfulness.tw.
75. (distraction adj3 therap*).tw.
76. self regulation training.tw.
77. 1 or 2 or 3 or 4 or 5 or 6
78. 7 or 8 or 9 or 10 or 11 or 12
79. 13 or 14 or 15 or 16 or 17 or 18 or 19 or 20 or 21 or 22 or 23 or 24 or 25 or 26 or 27 or 28 or 29 or 30 or 31 or 32 or 33 or 34 or 35 or 36 or 37 or 38 or 39 or 40 or 41 or 42 or 43 or 44 or 45

80. 46 or 47 or 48 or 49 or 50 or 51 or 52 or 53 or 54 or 55 or 56
81. 57 or 58 or 59 or 60 or 61 or 62 or 63 or 64 or 65 or 66 or 67 or 68 or 69 or 70 or 71 or 72 or 73 or 74 or 75 or 76
82. 77 and 78 and 79 and 80 and 81

EMBASE via OVID
1. exp Dentistry/
2. (dental* or dentist*).ti,ab.
3. (oral adj5 surg*).ti,ab.
4. (orthodontic* or pulpotom* or pulpect* or endodont* or "pulp cap*").mp.
5. ((dental or tooth or teeth) adj5 (fill* or restor* or extract* or remov* or "cavity prep*" or caries or carious or decay*)).mp.
6. (root canal and (therap* or treat*)).mp.
7. child/
8. Infant/
9. adolescent/
10. Pediatrics/
11. Dental care for children/
12. (child* or adolescent* or kid or kids or youth* or youngster* or minor or minors or teen* or preteen* or pre-teen* or juvenile* or "young adult*" or "young person" or "young people" or p?ediatric* or student* or pupil or pupils or boy or boys or girl or girls or under 18* or under eighteen* or underage).ti,ab,kw.
13. Pain, Postoperative/
14. (postoperative adj4 pain*).ti,ab,kw.
15. (post-operative adj4 pain*).ti,ab,kw.
16. post-operative-pain*.ti,ab,kw.
17. (post* adj4 pain*).ti,ab,kw.
18. (postoperative adj4 analgesi*).ti,ab,kw.
19. (post-operative adj4 analgesi*).ti,ab,kw.
20. post-operative analgesi*.ti,ab,kw.
21. (post-surgical adj4 pain*).ti,ab,kw.
22. (post surgical adj4 pain*).ti,ab,kw.
23. (pain* adj4 after surg*).ti,ab,kw.
24. (pain* adj4 after operat*).ti,ab,kw.
25. (pain* adj4 follow* operat*).ti,ab,kw.
26. (pain* adj4 follow* surg*).ti,ab,kw.
27. (pain adj1 operati*).ti,ab,kw.
28. (intra adj procedur* adj pain).ti,ab,kw.
29. (intraoperative adj4 pain*).ti,ab,kw.
30. (intra-operative adj4 pain*).ti,ab,kw.
31. intra-operative-pain*.ti,ab,kw.
32. (intraoperative adj4 analgesi*).ti,ab,kw.
33. (intra-operative adj4 analgesi*).ti,ab,kw.
34. intra-operative analgesi*.ti,ab,kw.
35. (intra-surgical adj4 pain*).ti,ab,kw.
36. (intrasurgical adj4 pain*).ti,ab,kw.
37. (pain* adj4 after surg*).ti,ab,kw.
38. (pain* adj4 after operat*).ti,ab,kw.
39. (pain* adj4 follow* operat*).ti,ab,kw.
40. (pain* adj4 follow* surg*).ti,ab,kw.

41. (surg* adj1 pain).ti,ab,kw.
42. (("post surg*" or post-surg*) and (pain* or discomfort)).ti,ab,kw.
43. (analgesi* adj4 surg*).ti,ab,kw.
44. (analgesi* adj4 operat*).ti,ab,kw.
45. (sore* or hurt* or ache* or aching or discomfort* or uncomfort* or tender* or throb*).mp. [mp=title, abstract, heading word, drug trade name, original title, device manufacturer, drug manufacturer, device trade name, keyword, floating subheading word, candidate term word]
46. An?esthetics, Local/
47. An?esthesia, Local/
48. (local adj5 (anesthetic* or anaesthetic* or anesthesia or anaesthesia)).mp.
49. Lidocaine/
50. (lidocaine or lignocaine or xylocaine).mp.
51. Carticaine/
52. (carticain* or articain*).mp.
53. Prilocaine/
54. (prilocain* or citanest* or propitocain* or xylonest).mp.
55. Bupivacaine/
56. (bupivacain* or buvacaina or carbostesin or dolanaest or marcain* or sensorcain* or svedocain*).mp.
57. Analgesi*.ti,ab,kw.
58. (anti-inflammatory or nitrous oxide sedation).mp. [mp=title, abstract, heading word, drug trade name, original title, device manufacturer, drug manufacturer, device trade name, keyword, floating subheading word, candidate term word]
59. (nonsteroidal anti-inflammatory agent* or anti inflammatory agent* or nonsteroidal antiinflammatory agent* or non steroidal antiinflammatory agent* or Nonsteroidal analgesic* or anti-inflammator* or aspirin-like agent* or NSAID*).ti,ab,kw.
60. (ibuprofen or brufen).ti,ab,kw.
61. (acetaminophen or paracetamol).ti,ab,kw.
62. pain management/
63. (pain adj3 management).tw.
64. psychotherapy/
65. psychotherap*.tw.
66. behavior therapy/
67. (behavi?r adj3 therap*).tw.
68. cognitive therapy/
69. (cognitive adj3 therap*).tw.
70. (relax* adj3 (technique* or therap*)).tw.
71. exp mind body therapies/
72. guided imagery.tw.
73. "imagery (Psychotherapy)"/
74. mindfulness.tw.
75. (distraction adj3 therap*).tw.
76. self regulation training.tw.
77. 1 or 2 or 3 or 4 or 5 or 6
78. 7 or 8 or 9 or 10 or 11 or 12
79. 13 or 14 or 15 or 16 or 17 or 18 or 19 or 20 or 21 or 22 or 23 or 24 or 25 or 26 or 27 or 28 or 29 or 30 or 31 or 32 or 33 or 34 or 35 or 36 or 37 or 38 or 39 or 40 or 41 or 42 or 43 or 44 or 45

80. 46 or 47 or 48 or 49 or 50 or 51 or 52 or 53 or 54 or 55 or 56
81. 57 or 58 or 59 or 60 or 61 or 62 or 63 or 64 or 65 or 66 or 67 or 68 or 69 or 70 or 71 or 72 or 73 or 74 or 75 or 76
82. 77 and 78 and 79 and 80 and 81

PsycINFO via OVID
1. exp Dentistry/
2. (dental* or dentist*).ti,ab.
3. (oral adj5 surg*).ti,ab.
4. (orthodontic* or pulpotom* or pulpect* or endodont* or "pulp cap*").mp.
5. ((dental or tooth or teeth) adj5 (fill* or restor* or extract* or remov* or "cavity prep*" or caries or carious or decay*)).mp.
6. (root canal and (therap* or treat*)).mp.
7. child/
8. Infant/
9. adolescent/
10. Pediatrics/
11. Dental care for children/
12. (child* or adolescent* or kid or kids or youth* or youngster* or minor or minors or teen* or preteen* or pre-teen* or juvenile* or "young adult*" or "young person" or "young people" or p?ediatric* or student* or pupil or pupils or boy or boys or girl or girls or under 18* or under eighteen* or underage).ti,ab,kw.
13. Pain, Postoperative/
14. (postoperative adj4 pain*).ti,ab,kw.
15. (post-operative adj4 pain*).ti,ab,kw.
16. post-operative-pain*.ti,ab,kw.
17. (post* adj4 pain*).ti,ab,kw.
18. (postoperative adj4 analgesi*).ti,ab,kw.
19. (post-operative adj4 analgesi*).ti,ab,kw.
20. post-operative analgesi*.ti,ab,kw.
21. (post-surgical adj4 pain*).ti,ab,kw.
22. (post surgical adj4 pain*).ti,ab,kw.
23. (pain* adj4 after surg*).ti,ab,kw.
24. (pain* adj4 after operat*).ti,ab,kw.
25. (pain* adj4 follow* operat*).ti,ab,kw.
26. (pain* adj4 follow* surg*).ti,ab,kw.
27. (pain adj1 operati*).ti,ab,kw.
28. (intra adj procedur* adj pain).ti,ab,kw.
29. (intraoperative adj4 pain*).ti,ab,kw.
30. (intra-operative adj4 pain*).ti,ab,kw.
31. intra-operative-pain*.ti,ab,kw.
32. (intraoperative adj4 analgesi*).ti,ab,kw.
33. (intra-operative adj4 analgesi*).ti,ab,kw.
34. intra-operative analgesi*.ti,ab,kw.
35. (intra-surgical adj4 pain*).ti,ab,kw.
36. (intrasurgical adj4 pain*).ti,ab,kw.
37. (pain* adj4 after surg*).ti,ab,kw.
38. (pain* adj4 after operat*).ti,ab,kw.
39. (pain* adj4 follow* operat*).ti,ab,kw.
40. (pain* adj4 follow* surg*).ti,ab,kw.

41. (surg* adj1 pain).ti,ab,kw.
42. (("post surg*" or post-surg*) and (pain* or discomfort)).ti,ab,kw.
43. (analgesi* adj4 surg*).ti,ab,kw.
44. (analgesi* adj4 operat*).ti,ab,kw.
45. (sore* or hurt* or ache* or aching or discomfort* or uncomfort* or tender* or throb*).mp. [mp=title, abstract, heading word, table of contents, key concepts, original title, tests & measures, mesh]
46. An?esthetics, Local/
47. An?esthesia, Local/
48. (local adj5 (anesthetic* or anaesthetic* or anesthesia or anaesthesia)).mp.
49. Lidocaine/
50. (lidocaine or lignocaine or xylocaine).mp.
51. Carticaine/
52. (carticain* or articain*).mp.
53. Prilocaine/
54. (prilocain* or citanest* or propitocain* or xylonest).mp.
55. Bupivacaine/
56. (bupivacain* or buvacaina or carbostesin or dolanaest or marcain* or sensorcain* or svedocain*).mp.
57. Analgesi*.ti,ab,kw.
58. (anti-inflammatory or nitrous oxide sedation).mp. [mp=title, abstract, heading word, table of contents, key concepts, original title, tests & measures, mesh]
59. (nonsteroidal anti-inflammatory agent* or anti inflammatory agent* or nonsteroidal antiinflammatory agent* or non steroidal antiinflammatory agent* or Nonsteroidal analgesic* or anti-inflammator* or aspirin-like agent* or NSAID*).ti,ab,kw.
60. (ibuprofen or brufen).ti,ab,kw.
61. (acetaminophen or paracetamol).ti,ab,kw.
62. pain management/
63. (pain adj3 management).tw.
64. psychotherapy/
65. psychotherap*.tw.
66. behavior therapy/
67. (behavi?r adj3 therap*).tw.
68. cognitive therapy/
69. (cognitive adj3 therap*).tw.
70. (relax* adj3 (technique* or therap*)).tw.
71. exp mind body therapies/
72. guided imagery.tw.
73. "imagery (Psychotherapy)"/
74. mindfulness.tw.
75. (distraction adj3 therap*).tw.
76. self regulation training.tw.
77. 1 or 2 or 3 or 4 or 5 or 6
78. 7 or 8 or 9 or 10 or 11 or 12
79. 13 or 14 or 15 or 16 or 17 or 18 or 19 or 20 or 21 or 22 or 23 or 24 or 25 or 26 or 27 or 28 or 29 or 30 or 31 or 32 or 33 or 34 or 35 or 36 or 37 or 38 or 39 or 40 or 41 or 42 or 43 or 44 or 45
80. 46 or 47 or 48 or 49 or 50 or 51 or 52 or 53 or 54 or 55 or 56

81. 57 or 58 or 59 or 60 or 61 or 62 or 63 or 64 or 65 or 66 or 67 or 68 or 69 or 70 or 71 or 72 or 73 or 74 or 75 or 76
82. 77 and 78 and 79 and 80 and 81

Cochrane Library (Wiley)
#1 DENTISTRY
#2 dental* or dentist*
#3 oral surg*
#4 orthodontic* or pulpotom* or pulpect* or endodont* or "pulp cap*"
#5 fill* or restor* or extract* or remov* or "cavity prep*" or caries or carious or decay*
#6 root canal and (therap* or treat*)
#7 #1 or #2 or #3 or #4 or #5 or #6
#8 child
#9 Infant
#10 adolescent
#11 Pediatric* or paediatric*
#12 Dental care for children
#13 child* or adolescent* or kid or kids or youth* or youngster* or minor or minors or teen* or preteen* or pre-teen* or juvenile* or "young adult*" or "young person" or "young people" or p?ediatric* or student* or pupil or pupils or boy or boys or girl or girls or under 18* or under eighteen* or underage
#14 #8 or #9 or #10 or #11 or #12 or #13
#15 Pain, Postoperative
#16 postoperative pain* or post-operative pain* or post* pain*
#17 postoperative analgesi* or post-operative analgesi* or post* analgesi*
#18 postsurgical pain* or post-surgical pain*
#19 pain* after surg* or pain* after operat* or pain* follow* operat* or pain* follow* surg*
#20 Pain, intraoperative
#21 intraoperative pain* or intra-operative pain* or intra* pain*
#22 intraoperative analgesi* or intra-operative analgesi* or intra* analgesi*
#23 intrasurgical pain* or intra-surgical pain*
#24 sore* or hurt* or ache* or aching or discomfort* or uncomfort* or tender* or throb*
#25 #15 or #16 or #17 or #18 or #19 or #20 or #21 or #22 or #23 or #24
#26 Analgesi* or anti-inflammatory or nitrous oxide sedation
#27 nonsteroidal anti-inflammatory agent* or anti inflammatory agent* or nonsteroidal antiinflammatory agent* or non steroidal antiinflammatory agent* or Nonsteroidal analgesic* or anti-inflammator* or aspirin-like agent* or NSAID*
#28 ibuprofen or brufen or acetaminophen or paracetamol
#29 pain management
#30 psychotherap*
#31 behavior therap* or cognitive therap* or mind body therap*
#32 #26 or #27 or #28 or #29 or #30 or #31
#33 local anesthetic* or local anaesthetic* or local anesthesia or local anaesthesia
#34 lidocain* or lignocain* or xylocain* or carticain* or articain* or prilocain* or citanest* or propitocain* or xylonest or bupivacain* or buvacaina or carbostesin or dolanaest or marcain* or sensorcain* or svedocain*
#35 #33 or #34

#36 #7 and #14 and #25 and #32 and #35

WHO International Clinical Trials Registry Platform
sed* or analgesi* or behavio?r management or behavio?r therapy and child* and dental

Clinical Trials.gov
Keywords: child and dental
Interventions: analgesics
Keywords: child and dental
Interventions: sedation
Keywords: child and dental
Interventions: behaviour management

Pubmed (Randomized Controlled Trial)
((((Dentistry or oral surg* or orthodontic* or pulpotom* or pulpect* or endodont* or "pulp cap*" or fill* or restor* or extract* or remov* or "cavity prep*" or caries or carious or decay* or root canal and (therap* or treat*)) AND (child* or adolescent* or kid or kids or youth* or young* or minor or minors or teen* or preteen* or pre-teen* or juvenile* or p?ediatric*)) AND (postoperative pain* or post-operative pain* or post* pain* or postoperative analgesi* or post-operative analgesi* or post* analgesi* or postsurgical pain* or post-surgical pain* or pain* after surg* or pain* after operat* or pain* follow* operat* or pain* follow* surg* or intraoperative pain* or intra-operative pain* or intra* pain* or intraoperative analgesi* or intra-operative analgesi* or intra* analgesi* or intrasurgical pain* or intra-surgical pain* or sore* or hurt* or ache* or aching or discomfort* or uncomfort* or tender* or throb*)) AND (Analgesi* or anti-inflammatory or nitrous oxide sedation or nonsteroidal anti-inflammatory agent* or anti inflammatory agent* or nonsteroidal antiinflammatory agent* or non steroidal antiinflammatory agent* or Nonsteroidal analgesic* or anti-inflammator* or aspirin-like agent* or NSAID* or ibuprofen or brufen or acetaminophen or paracetamol or pain management or psychotherap* or behavior therap* or cognitive therap* or mind body therap*)) AND (Local anesthetic* OR local anaesthetic* OR local anesthesia OR local anaesthesia OR lidocain* OR lignocain* OR xylocain* OR carticain* OR articain* OR prilocain* OR citanest* OR propitocain* OR xylonest OR bupivacain* OR buvacaina OR carbostesin OR dolanaest OR marcain* OR sensorcain* OR svedocain*)

Web of Science
#1 (DENTISTRY)
#2 (oral surg*
#3 (orthodontic* or pulpotom* or pulpect* or endodont* or "pulp cap*")
#4 (fill* or restor* or extract* or remov* or "cavity prep*" or caries or carious or decay*)
#5 (root canal and (therap* or treat*))
#6 #5 OR #4 OR #3 OR #2 OR #1
#7(child* or adolescent* or kid or kids or youth* or young* or minor or minors or teen* or preteen* or pre-teen* or juvenile* or p?ediatric*
#8 (Pain, Postoperative)
#9 (postoperative pain* or post-operative pain* or post* pain*)
#10 (postoperative analgesi* or post-operative analgesi* or post* analgesi*)

#11 (postsurgical pain* or post-surgical pain*)
#12 (pain* after surg* or pain* after operat* or pain* follow* operat* or pain* follow* surg*)
#13 (Pain, intraoperative)
#14 (intraoperative pain* or intra-operative pain* or intra* pain*)
#15 (intraoperative analgesi* or intra-operative analgesi* or intra* analgesi*)
#16 (intrasurgical pain* or intra-surgical pain*)
#17 (sore* or hurt* or ache* or aching or discomfort* or uncomfort* or tender* or throb*)
#18 #17 OR #16 OR #15 OR #14 OR #13 OR #12 OR #11 OR #10 OR #9 OR #8
#19 (Analgesi* or anti-inflammatory or nitrous oxide sedation)
#20 (nonsteroidal anti -inflammatory agent* or anti-inflammatory agent* or nonsteroidal antiinflammatory agent* or non steroidal antiinflammatory agent* or Nonsteroidal analgesic* or anti-inflammator* or aspirin-like agent* or NSAID*)
#21 (ibuprofen or brufen or acetaminophen or paracetamol)
#22(pain management or psychotherap* or behavior therap* or cognitive therap* or mind body therap*)
#23 #22 OR #21 OR #20 OR #19
#24 (local an?esthetic* or local an?esthesia)
#25 #24 AND #23 AND #18 AND #7 AND #6
