# Supplementary material for: Interventions to Reduce Intra-Operative and Post-Operative Pain Associated with Routine Dental Procedures in Children: A Systematic Review and Meta-Analysis
Source: Dent J (Basel). 2024 May 28;12(6):163. doi: 10.3390/dj12060163 (PMC11202832; doi:10.3390/dj12060163)
Supplement: Supplementary file 1 [file dentistry-12-00163-s001.zip › Supplementary file S2.pdf]

Supplementary file S2: Characteristics of included studies of the second systematic review

Agarwal 2017 [31]

Characteristics of included studies	
**Methods**	Study design: RCT, Parallel groups<br><br>Location: India<br><br>Setting: Department of Paedodontics and Preventive Dentistry, Institute of Dental Studies and Technologies
**Participants**	Children requiring local anaesthesia for the dental treatment<br><br>Sample size: 120<br><br>Age: 3-14 years old<br><br>Mean age: 8.8 years old
**Interventions**	Group A: EMLA (lidocaine 2.5% and prilocaine 2.5%) cream group without Audio Visual (AV) aids (n=30)<br><br>Group B: EMLA cream group with AV aids (n=30)<br><br>Group C: Benzocaine (20%) gel without AV aids (n=30)<br><br>Group D: Benzocaine gel with AV aids (n=30)<br><br>Sony Vaio laptop with earphones used as AV aids and DVD used were nursery rhymes and cartoon movies
**Outcomes**	Pain during LA: 0-10 Visual Analogue Scale

Risk of bias		
**Bias**	**Authors' judgement**	**Support for judgement**
**Random sequence generation**	Unclear risk	Participants were randomly allocated
**Allocation concealment**	Unclear risk	Allocation concealment method was not reported
**Blinding of participants and personnel for all outcomes**	High risk	It was not possible to blind the operators/participants to the intervention

**Blinding of outcome assessors for all outcomes**	Unclear risk	It is unclear whether assessor was blind
**Incomplete outcome data for all outcomes**	Low risk	The authors evaluated all included participants
**Selective outcome reporting**	Low risk	The authors reported all expected outcomes
**Other sources of bias**	Low risk	No other bias

Santos 2020 [39]

**Characteristics of included studies**	
**Methods**	Study design: RCT, Parallel groups<br><br>Location: Brazil<br><br>Setting: The Pediatric Postgraduate Clinic of Federal University of Santa Catarina
**Participants**	Children requiring extraction of primary molars<br><br>Sample size: 48<br><br>Age: 5-10 years old<br><br>Mean age: 7.17 years old
**Interventions**	Group A (Control): received placebo solution 1 hour before LA (n=16)<br>Group B: received paracetamol 200 mg/mL 1 hour before LA (n=16)<br>Group C: received ibuprofen 100 mg/mL 1 hour before LA (n=16)<br>All analgesics were taken orally
**Outcomes**	Pain after extraction at 2, 6, 24 hours: 0-100 Visual Analogue Scale

**Risk of bias**		
**Bias**	**Authors' judgement**	**Support for judgement**
**Random sequence generation**	Low risk	A randomised block design with permuted blocks of 4 and 6 patients each was used
**Allocation concealment**	Low risk	“Allocation was concealed with a pre-specified computer-generated randomization list, placed in numbered opaque sealed envelopes by a person not involved on the research”
**Blinding of participants and personnel for**	Low risk	Participants and operator were blind

**all outcomes**		
**Blinding of outcome assessors for all outcomes**	Low risk	Assessor was blind
**Incomplete outcome data for all outcomes**	Low risk	The authors evaluated all included participants
**Selective outcome reporting**	Low risk	The trial authors reported all expected outcomes
**Other sources of bias**	Low risk	No other bias

Ramirez-Carrasco 2017 [32]

**Characteristics of included studies**	
**Methods**	Study design: RCT, Parallel groups<br><br>Location: Mexico<br><br>Setting: The Pediatric Dentistry Clinic at the Autonomous University of San Luis Potos
**Participants**	Children requiring local anaesthesia for the dental treatment for the first time<br><br>Sample size: 40<br><br>Age: 5-9 years old<br><br>Mean age: 7.5 years old
**Interventions**	Group A (Control): children were told to use headphones to block out the dental drill's noise. No sound was transmitted (n=20).<br><br>Group B (Case): children were listed to a classic directive hypnosis intervention (n=20).
**Outcomes**	Pain during LA: 0-10 Face, Legs, Activity, Cry, Consolability scale

**Risk of bias**		
**Bias**	**Authors' judgement**	**Support for judgement**
**Random sequence generation**	Unclear risk	Participants were randomly assigned
**Allocation concealment**	Unclear risk	Allocation concealment method was not reported
**Blinding of participants and personnel for all outcomes**	High risk	It was not possible to blind the operators/participants to the intervention

**Blinding of outcome assessors for all outcomes**	Low risk	Assessors were blind
**Incomplete outcome data for all outcomes**	Low risk	The authors evaluated all included participants
**Selective outcome reporting**	Low risk	The authors reported all expected outcomes
**Other sources of bias**	Low risk	No other bias

Versloot 2008 [24]

**Characteristics of included studies**	
**Methods**	Study design: RCT, Parallel groups<br><br>Location: Netherlands<br><br>Setting: Secondary dental care practice specialised in treating children
**Participants**	Children requiring local anaesthesia for two subsequent treatment sessions<br><br>Sample size: 147<br><br>Age: 4-11 years old<br><br>Mean age: 6.4 years old
**Interventions**	Group A (Control): received traditional syringe injection via infiltration for maxillary teeth and IANB for mandibular teeth (N=76)<br><br>Group B (Case): received Wand injection via infiltration for maxillary teeth and periodontal ligament for mandibular teeth (N=71)
**Outcomes**	Pain during LA: 0-10 Modified version of the visual analogue scale<br><br>Anxiety during dental treatment: 1-5 the Dental Subscale of the Children’s Fear Survey Schedule CFSS-DS

**Risk of bias**		
**Bias**	**Authors' judgement**	**Support for judgement**
**Random sequence generation**	Low risk	Randomisation list generated by SPSS (SPSS Inc, 12.0, Chicago, USA)
**Allocation concealment**	Unclear risk	Allocation concealment method was not reported
**Blinding of participants and personnel for**	High risk	It was not possible to blind the operators/participants to the intervention

**all outcomes**		
**Blinding of outcome assessors for all outcomes**	High risk	All treatments were videotaped and analysed by two independent observers
**Incomplete outcome data for all outcomes**	Unclear risk	"For 20 children only their first treatment session could be included due to rescheduling of the second appointment"
**Selective outcome reporting**	Low risk	The authors reported all expected outcomes
**Other sources of bias**	Low risk	No other bias

Obadiah 2020 [38]

**Characteristics of included studies**	
**Methods**	Study design: RCT, Parallel groups<br><br>Location: India<br><br>Setting: Saveetha Dental College and Hospitals, Saveetha Institute of Medical and Technical sciences, Saveetha University
**Participants**	Children requiring local anaesthesia for extraction or pulpotomy<br><br>Sample size: 60<br><br>Age: 4-13 years old<br><br>Mean age: 8.43 years old
**Interventions**	Group A (Case):<br>Children were provided with the Bubble toy and deep breathing exercise was taught to the children (n=30)<br><br>Group B (Control): children were not taught about this breathing exercise and were not provided with any soap solutions (n=30)
**Outcomes**	Pain during LA: 0-10 Wong-Baker FACES scale<br><br>Anxiety at dental examination and during LA: 1-5 the Facial Index Scale

**Risk of bias**		
**Bias**	**Authors' judgement**	**Support for judgement**
**Random sequence generation**	Unclear risk	Participants were randomly allocated
**Allocation concealment**	Unclear risk	Method of allocation concealment was not reported
**Blinding of participants and personnel for**	High risk	It was not possible to blind the operators/participants to the intervention

**all outcomes**		
**Blinding of outcome assessors for all outcomes**	Unclear risk	It is unclear whether assessors were blind
**Incomplete outcome data for all outcomes**	Low risk	The authors evaluated all included participants
**Selective outcome reporting**	Low risk	The authors reported all expected outcomes
**Other sources of bias**	Low risk	No other bias

Baghdadi 1999 [45]

**Characteristics of included studies**	
**Methods**	Study design: RCT, Crossover<br><br>Location: Syria<br><br>Setting: Department of Pediatric Dentistry, Damascus University
**Participants**	Children requiring local anaesthesia for restoring of two primary/permanent antimere molars with lesions of similar size<br><br>Sample size: 28<br><br>Age: 6-12 years old<br><br>Mean age: 10.21 years old
**Interventions**	Group A (Control): received conventional LA (2% lidocaine with 1:80,000 epinephrine and restoration<br><br>Group B (Experimental): received Electronic dental anaesthesia (EDA) and restoration<br><br>EDA is a device that provides anaesthesia but with no needles and injections and it works on the gate control theory of pain
**Outcomes**	Pain during stages of restoration: 0-3 the Sound, Eye, and Motor (SEM) scale and 0-3 Color Scale

**Risk of bias**		
**Bias**	**Authors' judgement**	**Support for judgement**
**Random sequence generation**	Unclear risk	Participants were randomly divided
**Allocation concealment**	Unclear risk	Allocation concealment method was not reported
**Blinding of participants and personnel for**	High risk	It was not possible to blind the operators/participants to the intervention

**all outcomes**		
**Blinding of outcome assessors for all outcomes**	Unclear risk	It is unclear whether assessor was blind
**Incomplete outcome data for all outcomes**	Low risk	The authors evaluated all included participants
**Selective outcome reporting**	Low risk	The authors reported all expected outcomes
**Other sources of bias**	Low risk	No other bias

Smolarek 2020 [66]

**Characteristics of included studies**	
**Methods**	Study design: RCT, Parallel groups<br><br>Location: Brazil<br><br>Setting: The dental practice office at an elementary school called Integral Care Centre for Child and Adolescent and paediatric dental clinics from the Department of Dentistry at Ponta Grossa State University (UEPG), Ponta Grossa, Parana
**Participants**	Children requiring local anaesthesia for restoring of the upper posterior teeth<br><br>Sample size: 105<br><br>Age: 5-8 years old<br>Mean age: 6.56 years old
**Interventions**	Group A: received conventional anaesthesia (CA) (n=35)<br><br>Group B: received vibrational anaesthesia (VBA) using DentalVibe (n=35)<br><br>Group C: received computer-controlled local anaesthesia delivery (CCLAD) (n=35)
**Outcomes**	Pain during LA: 0-10 Wong-Baker FACES, 0-10 Visual Analogue Scales for pain<br><br>Anxiety before treatment, at dental office and immediately after LA: 0-8 The Venham Picture Test modified (VPTm) Scale

**Risk of bias**		
**Bias**	**Authors' judgement**	**Support for judgement**
**Random sequence generation**	Low risk	Computer-generated tables with blocked randomisation were used
**Allocation concealment**	Low risk	“accomplished by distributing the obtained codes in numbered black opaque envelopes”

**Blinding of participants and personnel for all outcomes**	High risk	It was not possible to blind the operators/participants to the intervention
**Blinding of outcome assessors for all outcomes**	Unclear risk	It is unclear whether assessors were blind
**Incomplete outcome data for all outcomes**	Low risk	The authors evaluated all included participants
**Selective outcome reporting**	Low risk	The authors reported all expected outcomes
**Other sources of bias**	Low risk	No other bias

Massignan 2020 [37]

**Characteristics of included studies**		
**Methods**	Study design: RCT, Parallel groups<br><br>Location: Brazil<br><br>Setting: Pediatric Postgraduate Clinic of Federal University of Santa Catarina	
**Participants**	Children requiring local anaesthesia for extraction of primary molars<br><br>Sample size: 43<br><br>Age: 6-10 years old<br><br>Mean age: 7.42 years old	
**Interventions**	Group A (Control): received lidocaine 2% by infiltration (n=22)<br><br>Group B (Intervention): received articaine 4% by infiltration (n=21)	
**Outcomes**	Pain after extraction at 2 and 6 hours: 0-10 the Faces Pain Scale-Revised (FPS-R)	
**Risk of bias**		
**Bias**	**Authors' judgement**	**Support for judgement**
**Random sequence generation**	Low risk	“The randomisation sequence was generated using WebSite Randomization.com (http://www.randomization.com)”
**Allocation concealment**	Low risk	sequentially numbered, opaque, sealed envelopes were considered

**Blinding of participants and personnel for all outcomes**	Low risk	Participant and the clinician were blind
**Blinding of outcome assessors for all outcomes**	Low risk	Assessor was blind
**Incomplete outcome data for all outcomes**	Low risk	The authors evaluated all included participants
**Selective outcome reporting**	Low risk	The authors reported all expected outcomes
**Other sources of bias**	Low risk	No other bias

Smail-Faugeron 2019 [64]

**Characteristics of included studies**		
**Methods**	Study design: RCT, Parallel groups and Split mouth<br><br>Location: France<br><br>Setting: Paediatric dentistry departments of three French universities (Nice, Paris and Rennes)	
**Participants**	Children requiring local anaesthesia for restoring of 1st permanent molars<br><br>Sample size: 158<br><br>Age: 7-15 years old<br><br>Mean age: 9 years old for split mouth and 10.4 years old for parallel groups	
**Interventions**	Split mouth RCT: one permanent first molar was randomly allocated to the intraosseous anaesthesia (IOA) and the other permanent first molar belonging to the same dental arch in the same child was allocated to the conventional infiltration anaesthesia (CIA) (n=30).<br><br>Parallel-arm RCT: one patient with one permanent molar first was randomly allocated to one of the techniques (IOA or CIA) (n=128).	
**Outcomes**	Pain during LA and restoration: 0-10 Visual Analogue Scale	
**Risk of bias**		
**Bias**	**Authors' judgement**	**Support for judgement**
**Random sequence generation**	Low risk	A computer-generated, permuted-block randomisation sequence, with two block sizes randomly varied were used

**Allocation concealment**	Unclear risk	Allocation concealment method was not reported
**Blinding of participants and personnel for all outcomes**	High risk	Participants were blind but it was not possible to blind clinicians to the intervention.
**Blinding of outcome assessors for all outcomes**	Low risk	Assessors were blind
**Incomplete outcome data for all outcomes**	Low risk	The authors evaluated all included participants
**Selective outcome reporting**	Low risk	The authors reported all expected outcomes
**Other sources of bias**	Low risk	No other bias

Alanazi 2019 [58]

**Characteristics of included studies**	
**Methods**	Study design: RCT, Crossover<br><br>Location: Saudi Arabia<br><br>Setting: Department of Paediatric Dentistry, Riyadh Elm University
**Participants**	Children requiring bilateral maxillary buccal infiltration analgesia for the dental treatment in the posterior teeth<br><br>Sample size: 60<br><br>Age: 6-7 years old<br><br>Mean age: 6.57 years old
**Interventions**	Group A (Control): received traditional LA via maxillary buccal infiltration<br><br>Group B (Test): received traditional LA via maxillary buccal infiltration and the cold and vibration device (Buzzing device as distraction)
**Outcomes**	Pain during LA: 0-10 Wong-Baker FACES scale

**Risk of bias**		
**Bias**	**Authors' judgement**	**Support for judgement**
**Random sequence generation**	Unclear risk	Participants were randomly divided
**Allocation concealment**	Unclear risk	Allocation concealment method was not reported

**Blinding of participants and personnel for all outcomes**	High risk	It was not possible to blind the operators/participants to the intervention
**Blinding of outcome assessors for all outcomes**	High risk	Assessor was not blind to the treatment
**Incomplete outcome data for all outcomes**	Low risk	The authors evaluated all included participants
**Selective outcome reporting**	Low risk	The authors reported all expected outcomes
**Other sources of bias**	Low risk	No other bias

Versloot 2005 [23]

**Characteristics of included studies**	
**Methods**	Study design: RCT, Parallel groups<br><br>Location: Netherlands<br><br>Setting: A specialist clinic
**Participants**	Children requiring local anaesthesia for the dental treatment<br><br>Sample size: 125<br><br>Age: 4-11 years old<br><br>Mean age: 6.2 years old
**Interventions**	Group A: received traditional LA via infiltration for maxillary teeth and IANB for mandibular teeth (n=58)<br><br>Group B: received Wand LA via infiltration for maxillary teeth and periodontal ligament for mandibular teeth (n=67)
**Outcomes**	Pain during LA: 0-10 Modified version of the visual analogue scale<br><br>Anxiety during dental treatment: 1-5 the Dental Subscale of the Children's Fear Survey Schedule CFSS-DS

**Risk of bias**		
**Bias**	**Authors' judgement**	**Support for judgement**
**Random sequence generation**	Low risk	A randomisation list generated by SPSS (SPSS, 11.0; Chicago, IL, USA) was used
**Allocation concealment**	Unclear risk	Allocation concealment method was not reported

**Blinding of participants and personnel for all outcomes**	High risk	It was not possible to blind the operators/participants to the intervention
**Blinding of outcome assessors for all outcomes**	High risk	"All treatments were videotaped and analysed by two independent observers"
**Incomplete outcome data for all outcomes**	Low risk	The authors evaluated all included participants
**Selective outcome reporting**	Low risk	The authors reported all expected outcomes
**Other sources of bias**	Low risk	No other bias

Arcari 2018 [34]

**Characteristics of included studies**	
**Methods**	Study design: RCT, Parallel groups<br><br>Location: Italy<br><br>Setting: Two private practice dental offices
**Participants**	Children requiring local anaesthesia for restoring of primary molars (class I/ II)<br><br>Sample size: 90<br><br>Age: 3-10 years old<br><br>Mean age: 6.2 years old
**Interventions**	Group A (Control): received nitrous oxide-oxygen (40% N_2O and 60% O_2) relative analgesia and LA (n=42)<br><br>Group B (Study): received nitrous oxide-oxygen (40% N_2O and 60% O_2) relative analgesia (n=48)
**Outcomes**	Pain during restoration: 0-10 Wong-Baker FACES scale

**Risk of bias**		
**Bias**	**Authors' judgement**	**Support for judgement**
**Random sequence generation**	Unclear risk	Participants were randomly assigned
**Allocation concealment**	Unclear risk	Method of allocation concealment not reported
**Blinding of participants and personnel for all outcomes**	High risk	It was not possible to blind the operators/participants to the intervention

**Blinding of outcome assessors for all outcomes**	High risk	Assessors were not blind
**Incomplete outcome data for all outcomes**	Low risk	The authors evaluated all included participants
**Selective outcome reporting**	Low risk	The authors reported all expected outcomes
**Other sources of bias**	Low risk	No other bias

Baghlaf 2015 [28]

**Characteristics of included studies**	
**Methods**	Study design: RCT, Parallel groups<br><br>Location: Saudi Arabia<br><br>Setting: The pediatric dentistry specialty clinics, King Abdulaziz University
**Participants**	Children requiring local anaesthesia for pulpotomy of primary mandibular 2nd molars<br><br>Sample size: 91<br><br>Age: 5-9 years old<br><br>Mean age: Not reported
**Interventions**	Group A: received traditional IANB (Inferior alveolar nerve block) (n=31)<br><br>Group B: received IANB with a CCLAD (Inferior alveolar nerve block with computer-controlled local anaesthetic delivery (CCLAD IANB)) (n=30)<br><br>Group C: received ILA with a CCLAD STA system (Intraligamental anaesthesia with computer-controlled local anesthetic delivery (CCLAD interligamental)) (n=30)
**Outcomes**	Pain during LA: 0-10 Wong-Baker FACES scale

**Risk of bias**		
**Bias**	**Authors' judgement**	**Support for judgement**
**Random sequence generation**	Unclear risk	Participants were randomly assigned using a block randomisation technique
**Allocation concealment**	Unclear risk	Allocation concealment method was not reported
**Blinding of participants and personnel for all outcomes**	High risk	It was not possible to blind the operators to the intervention

**Blinding of outcome assessors for all outcomes**	High risk	Assessors were not blind
**Incomplete outcome data for all outcomes**	Low risk	The authors reported on incomplete data "nine participants were excluded due to failure of the anaesthesia technique, or uncontrolled bleeding of the pulp, extraction or they refused to apply the rubber dam"
**Selective outcome reporting**	Low risk	The authors reported all expected outcomes
**Other sources of bias**	Low risk	No other bias

Sridhar 2019 [36]

**Characteristics of included studies**	
**Methods**	Study design: RCT, Parallel groups<br><br>Location: India<br><br>Setting: The Department of Paedodontics and Preventive Dentistry
**Participants**	Children requiring maxillary buccal infiltration anaesthesia for dental treatment<br><br>Sample size: 66<br><br>Age: 7-11 years old<br><br>Mean age: 8.75 years old
**Interventions**	Group A (control): not used the bubble breath exercise (n=33)<br><br>Group B: used the bubble breath exercise (n=33)
**Outcomes**	Pain during LA: 0-10 Wong-Baker FACES scale<br><br>Anxiety at the 1st appointment before dental examination and the 2nd appointment before local anaesthesia: 1-5 the Facial Image Scale

**Risk of bias**		
**Bias**	**Authors' judgement**	**Support for judgement**
**Random sequence generation**	Low risk	"Block randomization method with a block size of four was used"
**Allocation concealment**	Low risk	"The treatment group codes so generated (A or B) were entered into cards and placed in envelopes that were sequentially numbered. The envelopes were rendered opaque by covering the

		cards with aluminium foil and then sealed”
**Blinding of participants and personnel for all outcomes**	High risk	It was not possible to blind the operators/participants to the intervention
**Blinding of outcome assessors for all outcomes**	High risk	Assessors were not blind
**Incomplete outcome data for all outcomes**	Low risk	The authors evaluated all included participants
**Selective outcome reporting**	Low risk	The authors reported all expected outcomes
**Other sources of bias**	Low risk	No other bias

Attar 2015 [52]

**Characteristics of included studies**	
**Methods**	Study design: RCT, Split mouth<br><br>Location: Saudi Arabia<br><br>Setting: The paediatric clinic in the Department of Preventive Dentistry Riyadh Colleges of Dentistry and Pharmacy
**Participants**	Children requiring local anaesthesia for pulpotomy of two primary antimere molars<br><br>Sample size: 39<br><br>Age: 4-8 years old<br><br>Mean age: 6.27 years old
**Interventions**	Group A (Control): received treatment with the aid of audio-visual (AV) glasses<br><br>Group B (Exposure): received treatment with the aid of an iPad video game
**Outcomes**	Pain at 5 mins before LA, during LA and stages of pulpotomy and 5 mins post-operatively: 0-3 Wong-Baker FACES scale<br><br>Anxiety before dental treatment: 1-5 The Modified Dental Anxiety Scale (MDAS)

**Risk of bias**		
**Bias**	**Authors' judgement**	**Support for judgement**
**Random sequence generation**	Unclear risk	Participants were randomly allocated

**Allocation concealment**	Unclear risk	Allocation concealment method was not reported
**Blinding of participants and personnel for all outcomes**	High risk	It was not possible to blind the operators/participants to the intervention
**Blinding of outcome assessors for all outcomes**	Unclear risk	It is unclear whether assessor was blind
**Incomplete outcome data for all outcomes**	Low risk	The authors evaluated all included participants
**Selective outcome reporting**	Low risk	The authors reported all expected outcomes
**Other sources of bias**	Low risk	No other bias

Atabek 2015 [50]

**Characteristics of included studies**	
**Methods**	Study design: RCT, Crossover<br><br>Location: Turkey<br><br>Setting: The Department of Pedodontics, Faculty of Dentistry, Gazi University
**Participants**	Children requiring bilateral maxillary infiltration anaesthesia for restoring maxillary primary molars<br><br>Sample size: 50<br><br>Age: 8-12 years old<br><br>Mean age: 9 years old
**Interventions**	Group A (Control): received topical anaesthetic solution of 10 % lidocaine pump spray<br><br>Group B (Case): received three-in-one injection comfort system (ICS) which provides tissue retraction, illumination of the area, and pain blockage
**Outcomes**	Pain during LA: 0-10 Wong-Baker FACES scale

**Risk of bias**		
**Bias**	**Authors' judgement**	**Support for judgement**
**Random sequence generation**	Unclear risk	Randomisation method was not reported
**Allocation concealment**	Unclear risk	Allocation concealment method was not reported

**Blinding of participants and personnel for all outcomes**	High risk	It was not possible to blind the operators/participants to the intervention
**Blinding of outcome assessors for all outcomes**	Low risk	Assessor was blind
**Incomplete outcome data for all outcomes**	Low risk	The authors evaluated all included participants
**Selective outcome reporting**	Low risk	The authors reported all expected outcomes
**Other sources of bias**	Low risk	No other bias

Dak-Albab 2016 [54]

**Characteristics of included studies**	
**Methods**	Study design: RCT, Crossover<br><br>Location: Syria<br><br>Setting: Pediatric Dentistry department in the Dental College, Damascus University
**Participants**	Children requiring two mandibular nerve block analgesia for symmetric dental treatment<br><br>Sample size: 30<br><br>Age: 8-12 years old<br><br>Mean age: Not reported
**Interventions**	Technique A: received benzocaine 20% topical gel and LA<br><br>Technique B: received vibration using DentalVibe and LA
**Outcomes**	Pain during LA: 0-10 Face, Legs, Activity, Cry, Consolability scale

**Risk of bias**		
**Bias**	**Authors' judgement**	**Support for judgement**
**Random sequence generation**	Unclear risk	Participants were randomly allocated
**Allocation concealment**	Unclear risk	Allocation concealment method was not reported
**Blinding of participants and personnel for all outcomes**	High risk	It was not possible to blind the operators/participants to the intervention

**Blinding of outcome assessors for all outcomes**	Low risk	Assessor was blind
**Incomplete outcome data for all outcomes**	Low risk	The authors evaluated all included participants
**Selective outcome reporting**	Low risk	The authors reported all expected outcomes
**Other sources of bias**	Low risk	No other bias

Ram 2006 [48]

**Characteristics of included studies**	
**Methods**	Study design: RCT, Crossover<br><br>Location: Israel<br><br>Setting: Two established paediatric dental clinics in Jerusalem and Tel Aviv
**Participants**	Children requiring two local analgesia for similar operative procedures in the same arch<br><br>Sample size: 62<br><br>Age: 5-13 years old<br><br>Mean age: 8.4 years old
**Interventions**	Group A: received lidocaine HCl 2% with 1: 100 000 epinephrine<br><br>Group B: received articaine HCl 4% with 1: 200 000 epinephrine
**Outcomes**	Pain during LA: 0-10 Wong-Baker FACES scale

**Risk of bias**		
**Bias**	**Authors' judgement**	**Support for judgement**
**Random sequence generation**	Unclear risk	Participants were randomly allocated
**Allocation concealment**	Unclear risk	Allocation concealment method was not reported
**Blinding of participants and personnel for all outcomes**	Unclear risk	It is unclear whether participants and clinicians were blind
**Blinding of outcome assessors for all outcomes**	Low risk	Assessor was blind
**Incomplete outcome data for all outcomes**	Low risk	The authors evaluated all included participants
**Selective outcome reporting**	Low risk	The authors reported all expected outcomes

**Other sources of bias**	Unclear risk	The authors did not report which LA agent was given by infiltration or mandibular block injections

Garrocho-Rangel 2018 [56]

**Characteristics of included studies**	
**Methods**	Study design: RCT, Crossover<br><br>Location: Mexico<br><br>Setting: The paediatric dentistry clinic, San Luis Potosi University
**Participants**	Children requiring two local analgesia for restoring two upper or lower primary molars<br><br>Sample size: 36<br><br>Age: 5-8 years old<br><br>Mean age: 6.2 years old
**Interventions**	Group A (Control): received treatment without using the Video Eyeglasses/Earphones System (VEES)<br><br>Group B (Experimental): received treatment with using the VEES
**Outcomes**	Pain during LA and restoration: 0-10 Face, Legs, Activity, Cry, Consolability scale

**Risk of bias**		
**Bias**	**Authors' judgement**	**Support for judgement**
**Random sequence generation**	Low risk	A randomisation block scheme was used
**Allocation concealment**	Unclear risk	Allocation concealment method was not reported
**Blinding of participants and personnel for all outcomes**	High risk	It was not possible to blind the operators/participants to the intervention
**Blinding of outcome assessors for all outcomes**	High risk	Assessor was not blind
**Incomplete outcome data for all outcomes**	Low risk	The authors evaluated all included participants
**Selective outcome reporting**	Low risk	The authors reported all expected outcomes
**Other sources of bias**	Low risk	No other bias

Yildirim 2020 [60]

Characteristics of included studies	
**Methods**	Study design: RCT, Crossover<br><br>Location: Turkey<br><br>Setting: Faculty of Dentistry, Istanbul Okan University
**Participants**	Children requiring two mandibular nerve block analgesia for dental treatment their bilateral mandibular primary or permanent molars<br><br>Sample size: 60<br><br>Age: 6-12 years old<br><br>Mean age: 8.37 years old
**Interventions**	Group A (Control): received topical anaesthesia (TA) spray containing 10% lidocaine with a cotton pellet for 60s<br><br>Group B (Case): received Comfort-in™injection system (CIS) which uses the “liquid jet” system to inject the anaesthetic solution rapidly (one-third of a second) from a 0.15-mm hole with high pressure
**Outcomes**	Pain during LA: 0-5 Wong-Baker FACES scale and 0-10 Face, Legs, Activity, Cry, Consolability scale

Risk of bias		
**Bias**	**Authors' judgement**	**Support for judgement**
**Random sequence generation**	Low risk	A computer-assisted program was used
**Allocation concealment**	Low risk	“The operator was asked to select the side to do the first treatment before the researcher revealed the pre-anaesthesia method to be applied, to avoid possible operator bias”
**Blinding of participants and personnel for all outcomes**	High risk	It was not possible to blind the operators/participants to the intervention
**Blinding of outcome assessors for all outcomes**	Unclear risk	It is unclear whether assessor was blind
**Incomplete outcome data for all outcomes**	Low risk	The authors evaluated all included participants
**Selective outcome reporting**	Low risk	The authors reported all expected outcomes
**Other sources of bias**	Low risk	No other bias

Alinejhad 2018 [55]

Characteristics of included studies	
**Methods**	Study design: RCT, Crossover<br><br>Location: Iran<br><br>Setting: The Department of Pediatrics of the Faculty of Dentistry at Shahid Sadoughi University of Medical Sciences
**Participants**	Children requiring local anaesthesia for pulpotomy of primary mandibular 2nd molars<br><br>Sample size: 40<br><br>Age: 6-10 years old<br><br>Mean age: Not reported
**Interventions**	Group A: received 2% lidocaine with epinephrine 1:100,000 by IANB<br><br>Group B: received 4% articaine with epinephrine 1:100,000 by buccal infiltration
**Outcomes**	Pain during LA: 0-4 Visual Analogue Scale

Risk of bias		
**Bias**	**Authors' judgement**	**Support for judgement**
**Random sequence generation**	Unclear risk	Participants were randomly allocated
**Allocation concealment**	Unclear risk	Allocation concealment method not reported
**Blinding of participants and personnel for all outcomes**	High risk	It was not possible to blind the operators to the intervention
**Blinding of outcome assessors for all outcomes**	High risk	Assessors were not blind
**Incomplete outcome data for all outcomes**	Low risk	The authors evaluated all included participants
**Selective outcome reporting**	Low risk	The authors reported all expected outcomes
**Other sources of bias**	Low risk	No other bias

Gumus 2020 [59]

Characteristics of included studies	
**Methods**	Study design: RCT, Split mouth<br><br>Location: Turkey

	Setting: The Pediatric Dentistry Clinic of the Erciyes University, Faculty of Dentistry
**Participants**	Children requiring two local analgesia for dental treatment of bilateral maxillary primary molars<br><br>Sample size: 100<br><br>Age: 5-8 years old<br><br>Mean age: 6.5 years old for girls and 6.42 years old for boys
**Interventions**	Group A: received a cartridge containing 2 mL of LA solution was placed in the CALSET composite heater and warmed to body temperature (37 °C)<br><br>Group B: received a cartridge containing a LA solution was immersed in a 21 °C water bath, half an hour prior to the procedure
**Outcomes**	Pain during LA: 0-10 Wong-Baker FACES scale

**Risk of bias**		
**Bias**	**Authors' judgement**	**Support for judgement**
**Random sequence generation**	Low risk	"MS Excel 2013 (Microsoft Corporation, Redmond, WA, USA) software was used to randomly determine which side(right/left) of the maxilla was to be infiltrated with the anaesthetic solution and at which temperature (21 °C or 37 °C) in the first session"
**Allocation concealment**	Unclear risk	Allocation concealment method not reported
**Blinding of participants and personnel for all outcomes**	High risk	It was not possible to blind the operators to the intervention
**Blinding of outcome assessors for all outcomes**	Unclear risk	It is unclear whether assessors were blind
**Incomplete outcome data for all outcomes**	Low risk	The authors evaluated all included participants
**Selective outcome reporting**	Low risk	The authors reported all expected outcomes
**Other sources of bias**	Low risk	No other bias

Tung 2018 [35]

**Characteristics of included studies**	
**Methods**	Study design: RCT, Parallel groups

	Location: USA<br><br>Setting: Herman Ostrow school of dentistry, university of Southern California
**Participants**	Children requiring a maxillary infiltration injection or mandibular inferior alveolar block and long buccal anaesthesia for operative dental treatment<br><br>Sample size: 150<br><br>Age: 7-14 years old<br><br>Mean age: 11.1 years old for groups A and C and 10.7 years old for group B
**Interventions**	Group A: received an injection without stimulation (n=50)<br><br>Group B: received an injection with manual stimulation (n=50)<br><br>Group C: received an injection with Dental Vibe (n=50)
**Outcomes**	Pain during LA: 0-10 Wong-Baker FACES scale

**Risk of bias**		
**Bias**	**Authors' judgement**	**Support for judgement**
**Random sequence generation**	Low risk	“Using a table of randomly generated numbers, the subjects were assigned to one of three groups”
**Allocation concealment**	Low risk	Participants were randomised prior to attendance
**Blinding of participants and personnel for all outcomes**	High risk	It was not possible to blind the operators/participants to the intervention
**Blinding of outcome assessors for all outcomes**	High risk	Assessors were not blind
**Incomplete outcome data for all outcomes**	Low risk	The authors evaluated all included participants
**Selective outcome reporting**	Low risk	The authors reported all expected outcomes
**Other sources of bias**	Low risk	No other bias

Shilpapriya 2015 [53]

**Characteristics of included studies**	
**Methods**	Study design: RCT, Crossover

	Location: India<br><br>Setting: The Department of Pedodontics and Preventive Dentistry of Ragas Dental College and Hospital
**Participants**	Children requiring bilateral local anaesthesia for dental treatment<br><br>Sample size: 30<br><br>Age: 6-12 years old<br><br>Mean age: 7.5 years old
**Interventions**	Group A: received an injection without DentalVibe<br><br>Group B: received an injection with DentalVibe
**Outcomes**	Pain during LA: 0-10 Universal Pain Assessment Tool

**Risk of bias**		
**Bias**	**Authors' judgement**	**Support for judgement**
**Random sequence generation**	Unclear risk	Method of randomisation was not reported
**Allocation concealment**	Unclear risk	Method of allocation concealment not reported
**Blinding of participants and personnel for all outcomes**	High risk	It was not possible to blind the operators/participants to the intervention
**Blinding of outcome assessors for all outcomes**	Unclear risk	It is unclear whether assessors were blind
**Incomplete outcome data for all outcomes**	Low risk	The authors evaluated all included participants
**Selective outcome reporting**	Low risk	The authors reported all expected outcomes
**Other sources of bias**	Unclear risk	No population characteristics other than age

Alamoudi 2015 [27]

**Characteristics of included studies**	
**Methods**	Study design: RCT, Parallel groups<br><br>Location: Saudi Arabia<br><br>Setting: Faculty of Dentistry, King Abdulaziz University
**Participants**	Children requiring local anaesthesia for pulpotomy of primary mandibular 2nd molars

	Sample size: 91<br><br>Age: 5-9 years old<br><br>Mean age: Not reported
**Interventions**	Group A: received traditional IANB (Inferior alveolar nerve block) (n=31)<br><br>Group B: received IANB with a CCLAD (Inferior alveolar nerve block with computer-controlled local anaesthetic delivery (CCLAD IANB) (n=30)<br><br>Group C: received ILA with a CCLAD STA system (Intraligamental anaesthesia with computer-controlled local anaesthetic delivery) (n=30)
**Outcomes**	Pain during stages of pulpotomy: 1-4 the Sounds, Eyes, and Motor (SEM) scale

**Risk of bias**		
**Bias**	**Authors' judgement**	**Support for judgement**
**Random sequence generation**	Low risk	"Block randomisation technique was applied to assign participants to one of the three study groups"
**Allocation concealment**	Unclear risk	The authors referred to allocation concealment but did not explain the method
**Blinding of participants and personnel for all outcomes**	High risk	It was not possible to blind the operators/participants to the intervention
**Blinding of outcome assessors for all outcomes**	Low risk	Assessors was blind
**Incomplete outcome data for all outcomes**	Low risk	The authors evaluated all included participants
**Selective outcome reporting**	Low risk	The authors reported all expected outcomes
**Other sources of bias**	Unclear risk	No baseline characteristics reported

Mumtaz 2021 [20]

**Characteristics of included studies**	
**Methods**	Study design: RCT, Parallel groups<br><br>Location: India<br><br>Setting: The Department of Pedodontics and Preventive Dentistry

**Participants**	Children requiring local anaesthesia for extraction of mandibular primary molars<br><br>Sample size: 70<br><br>Age: 8-10 years old<br><br>Mean age: Not reported
**Interventions**	Group A: received 1.5ml of 2 % lignocaine with 1:100000 epinephrine via inferior alveolar nerve block (n=35)<br><br>Group B: received 1.5 ml of 4 % articaine with 1:10000 epinephrine via buccal and lingual infiltration (n=35)
**Outcomes**	Pain during extraction: 0-10 Visual Analogue Scale

**Risk of bias**		
**Bias**	**Authors' judgement**	**Support for judgement**
**Random sequence generation**	Unclear risk	Participants were randomly allocated
**Allocation concealment**	Unclear risk	Allocation concealment method was not reported
**Blinding of participants and personnel for all outcomes**	High risk	It was not possible to blind the operators/participants to the intervention
**Blinding of outcome assessors for all outcomes**	Unclear risk	It is unclear whether assessors were blind
**Incomplete outcome data for all outcomes**	Low risk	The authors evaluated all included participants
**Selective outcome reporting**	Low risk	The authors reported all expected outcomes
**Other sources of bias**	Unclear risk	No baseline characteristics reported

Abdelmoniem 2016 [30]

**Characteristics of included studies**	
**Methods**	Study design: RCT, Parallel groups<br><br>Location: Egypt<br><br>Setting: Pediatric Dentistry and Dental Public Health Department, Faculty of Oral and Dental Medicine, Cairo University
**Participants**	Children requiring inferior alveolar nerve block for extraction of mandibular primary molar<br><br>Sample size: 90

	Age: 4-9 years old<br><br>Mean age: 7.18 years old for group A and 7.02 years old for group B and 7.65 years old for group C
**Interventions**	Group A: received passive distraction by listening to the same song on headphones (n=30)<br><br>Group B: received active distraction by moving legs up and down alternatively as a game (n=30)<br><br>Group C: received passive-active distraction group (n=30)
**Outcomes**	Pain during LA: 0-10 Wong-Baker FACES scale

**Risk of bias**		
**Bias**	**Authors' judgement**	**Support for judgement**
**Random sequence generation**	Unclear risk	Participants were randomly allocated
**Allocation concealment**	Unclear risk	Allocation concealment method was not reported
**Blinding of participants and personnel for all outcomes**	High risk	It was not possible to blind the operators/participants to the intervention
**Blinding of outcome assessors for all outcomes**	Unclear risk	It was not reported
**Incomplete outcome data for all outcomes**	Low risk	The authors evaluated all included participants
**Selective outcome reporting**	Low risk	The authors reported all expected outcomes
**Other sources of bias**	Low risk	No other bias

Huet 2011 [25]

**Characteristics of included studies**	
**Methods**	Study design: RCT, Parallel groups<br><br>Location: France<br><br>Setting: Department of Pediatric Dentistry at Rennes University Hospital
**Participants**	Children requiring local anaesthesia for dental treatment

	Sample size: 29<br><br>Age: 7-12 years old<br><br>Mean age: Not reported
**Interventions**	Group A: received LA with hypnosis (n=14)<br><br>Group B: received LA without hypnosis (n=15)
**Outcomes**	Pain during LA: 0-10 Modified Objective Pain Score (mOPS) Scale<br><br>Anxiety at during the initial interview, on arrival in the waiting room, in the dentist's chair and at the time of the dental anaesthesia: 0-100 The Modified Yale Preoperative Anxiety Scale (mYPAS)

**Risk of bias**		
**Bias**	**Authors' judgement**	**Support for judgement**
**Random sequence generation**	Low risk	Participants were randomly allocated by lottery
**Allocation concealment**	Unclear risk	Allocation concealment method was not reported
**Blinding of participants and personnel for all outcomes**	High risk	It was not possible to blind operator/participants to the intervention
**Blinding of outcome assessors for all outcomes**	High risk	Assessor was not blind
**Incomplete outcome data for all outcomes**	Low risk	The authors reported on incomplete data "One child excluded because of un-usable data" from the intervention group
**Selective outcome reporting**	Low risk	The authors reported all expected outcomes
**Other sources of bias**	Low risk	No other bias

Bernhardt 2001 [22]

**Characteristics of included studies**	
**Methods**	Study design: RCT, Parallel groups<br><br>Location: USA<br><br>Setting: Department of Orthodontics, College of Dentistry
**Participants**	Children requiring separator placement for orthodontic treatment<br><br>Sample size: 41

	Age: 9-16 years old<br><br>Mean age: 12.1 years old for group A and 13.5 years old for group B and 12.8 years old for group C
**Interventions**	Group A: received 400 mg ibuprofen 1 hour preoperatively and 400 mg ibuprofen 6 hours after the initial dose (n=13)<br><br>Group B: received 400 mg ibuprofen 1 hour preoperatively and placebo 6 hours after the initial dose (n=14)<br><br>Group C: received placebo 1 hour preoperatively and 400 mg ibuprofen 6 hours after the initial dose (n=14)<br><br>All analgesics were taken orally
**Outcomes**	Pain after separator placement: 0-100 Visual Analogue scale

**Risk of bias**		
**Bias**	**Authors' judgement**	**Support for judgement**
**Random sequence generation**	Unclear risk	Participants were randomly allocated
**Allocation concealment**	Unclear risk	Allocation concealment method was not reported
**Blinding of participants and personnel for all outcomes**	Low risk	Operator and participants were blind to the intervention
**Blinding of outcome assessors for all outcomes**	Low risk	Assessors were blind
**Incomplete outcome data for all outcomes**	Low risk	The authors reported on incomplete data “22 of whom took additional medication and were excluded from the study. These 22 patients were evenly distributed among the 3 groups”
**Selective outcome reporting**	Low risk	The authors reported all expected outcomes
**Other sources of bias**	Low risk	No other bias

Law 2000 [21]

**Characteristics of included studies**	
**Methods**	Study design: RCT, Parallel groups<br><br>Location: USA<br><br>Setting: Department of Orthodontics, College of Dentistry

**Participants**	Children requiring separator placement for orthodontic treatment<br><br>Sample size: 63<br><br>Age: a maximum age of 16 years old<br><br>Mean age: 13 years old
**Interventions**	Group A: received 400 mg ibuprofen 1 hour preoperatively and placebo immediately after the appointment (n=22)<br><br>Group B: received placebo 1 hour preoperatively and 400 mg ibuprofen immediately after the appointment (n=19)<br><br>Group C: received placebo 1 hour preoperatively and placebo immediately after the appointment (n=22)<br><br>All analgesics were taken orally
**Outcomes**	Pain after separator placement: 0-100 Visual Analogue scale

**Risk of bias**		
**Bias**	**Authors' judgement**	**Support for judgement**
**Random sequence generation**	Unclear risk	Participants were randomly allocated
**Allocation concealment**	Unclear risk	Allocation concealment method was not reported
**Blinding of participants and personnel for all outcomes**	Low risk	Operator and participants were blind to the intervention
**Blinding of outcome assessors for all outcomes**	Low risk	Assessors were blind
**Incomplete outcome data for all outcomes**	Low risk	The authors reported on incomplete data “28 subjects did not receive separators at their next appointment and 17 subjects forgot to take the pretreatment dose before their appointment”
**Selective outcome reporting**	Low risk	The authors reported all expected outcomes
**Other sources of bias**	Low risk	No other bias

Alshami 2021 [41]

**Characteristics of included studies**	
**Methods**	Study design: RCT, Parallel groups<br><br>Location: Saudi Arabia<br><br>Setting: Princess Nourah bint Abdulrahman University’s dental clinic

**Participants**	Children requiring local anaesthesia for extraction of symptomatic primary molars<br><br>Sample size: 56<br><br>Age: 5-13 years old<br><br>Mean age: 9.4 years old
**Interventions**	Group A: received 7.5–15 ml/kg ibuprofen preoperatively (n=28)<br><br>Group B: received placebo preoperatively (n=28)<br><br>All analgesics were taken orally
**Outcomes**	Pain at baseline and after extraction at 3 and 24 hours: 0-10 Wong-Baker FACES scale<br><br>Anxiety at baseline and after extraction at 3 and 24 hours: 1-5 the Modified Child Dental Anxiety scale

**Risk of bias**		
**Bias**	**Authors' judgement**	**Support for judgement**
**Random sequence generation**	Unclear risk	Participants were randomly allocated
**Allocation concealment**	Low risk	“Once participants underwent consent, they were assigned an ID number which placed them in a randomised group”
**Blinding of participants and personnel for all outcomes**	Low risk	Operator and participants were blind to the intervention
**Blinding of outcome assessors for all outcomes**	High risk	Assessor was not blind
**Incomplete outcome data for all outcomes**	Low risk	The authors reported on incomplete data “One participant was removed from the analysis because they had three extractions, while the other participants had only one or two”
**Selective outcome reporting**	Low risk	The authors reported all expected outcomes
**Other sources of bias**	Low risk	No other bias

Helmy 2022 [44]

**Characteristics of included studies**	
**Methods**	Study design: RCT, Parallel groups<br><br>Location: Egypt

	Setting: The Pediatric Dentistry and Dental Public Health Department, Faculty of Dentistry, Alexandria University
**Participants**	Children requiring local anaesthesia for extraction of mandibular primary molars<br><br>Sample size: 50<br><br>Age: 5-7 years old<br><br>Mean age: 6.10 years old
**Interventions**	Group A: received Computer-controlled Intraligamentary anaesthesia (CC–ILA) (n=25)<br><br>Group B: received Inferior alveolar nerve block (IANB) (n=25)
**Outcomes**	Pain during LA and extraction: 1-4 the Sounds, Eyes, and Motor (SEM) scale

**Risk of bias**		
**Bias**	**Authors' judgement**	**Support for judgement**
**Random sequence generation**	Low risk	A computer–generated list of random numbers was used
**Allocation concealment**	Low risk	“Each child was given a serial number written in identical sheets of paper with the group to which each child is allocated and placed inside opaque envelopes carrying their respective names”
**Blinding of participants and personnel for all outcomes**	High risk	It was not possible to blind the operator to the intervention
**Blinding of outcome assessors for all outcomes**	Low risk	Assessor was blind
**Incomplete outcome data for all outcomes**	Low risk	The authors reported on incomplete data “One participant was removed from the analysis because they had three extractions, while the other participants had only one or two”
**Selective outcome reporting**	Low risk	The authors reported all expected outcomes
**Other sources of bias**	Low risk	No other bias

Vidigal 2021 [43]

**Characteristics of included studies**	
**Methods**	Study design: RCT, Parallel groups

	Location: Brazil<br><br>Setting: The Dental School, University of Sao Paulo
**Participants**	Children requiring local anaesthesia for extraction or pulpotomy of mandibular primary molars<br><br>Sample size: 52<br><br>Age: 3-5 years old<br><br>Mean age: Not reported
**Interventions**	Group A: received Tell-Show-Do Technique (TSD-T) (n=26)<br><br>Group B: received Hiding Dental-Needle Technique (HDN-T) (n=26)<br><br>LA was given by IANB with 1.8 ml of Lidocaine 2% with 1:100.000 epinephrine.
**Outcomes**	Pain during LA: 1-5 Wong-Baker FACES scale<br><br>Anxiety before and during LA: 1-5 the Facial Image scale

**Risk of bias**		
**Bias**	**Authors' judgement**	**Support for judgement**
**Random sequence generation**	Low risk	A statistical program MedCalc Software, version 12.4.0.0 was used
**Allocation concealment**	Low risk	“The sequence of numbers generated was distributed in opaque envelopes by an external researcher. The envelopes were opened only by the operator at the time of the block mandibular anesthesia.”
**Blinding of participants and personnel for all outcomes**	High risk	It was not possible to blind the operator/participants to the intervention
**Blinding of outcome assessors for all outcomes**	Low risk	Assessors were blind
**Incomplete outcome data for all outcomes**	Low risk	The authors evaluated all included participants
**Selective outcome reporting**	Low risk	The authors reported all expected outcomes
**Other sources of bias**	Low risk	No other bias

Ghaderi 2013 [49]

Characteristics of included studies	
**Methods**	Study design: RCT, Crossover<br><br>Location: Iran<br><br>Setting: Department of Pediatric Dentistry, Shiraz University of Medical Sciences
**Participants**	Children requiring bilateral buccal infiltration of local anaesthesia for extraction of maxillary primary canine on both sides<br><br>Sample size: 50<br><br>Age: 8-10 years old<br><br>Mean age: 8.94 years old
**Interventions**	Group A: received a topical anaesthetic agent (Benzocaine) on one side for 1 min and plus ice<br><br>Group B: received a topical anaesthetic agent (Benzocaine) on the other side for 1 min
**Outcomes**	Pain during LA: 0-100 Visual Analogue Scale and 0-3 the Sounds, Eyes, and Motor (SEM) scale

Risk of bias		
**Bias**	**Authors' judgement**	**Support for judgement**
**Random sequence generation**	Low risk	“Random number table have been used for block randomization. The number was chosen by tracing a line starting from random number till reaching to a block which was chosen as designated number.”
**Allocation concealment**	Unclear risk	Allocation concealment method was not reported
**Blinding of participants and personnel for all outcomes**	High risk	It was not possible to blind the operator/participants to the intervention
**Blinding of outcome assessors for all outcomes**	Low risk	Assessor was blind
**Incomplete outcome data for all outcomes**	Low risk	The authors evaluated all included participants
**Selective outcome reporting**	Low risk	The authors reported all expected outcomes
**Other sources of bias**	Unclear risk	No baseline characteristics reported

Jain 2021 [63]

Characteristics of included studies	
**Methods**	Study design: RCT, Split mouth<br><br>Location: India<br><br>Setting: Department of Paediatric and Preventive Dentistry, Sri Aurobindo College of Dentistry
**Participants**	Children requiring bilateral buccal infiltration of local anaesthesia for dental treatment of posterior maxillary teeth<br><br>Sample size: 30<br><br>Age: 5-10 years old<br><br>Mean age: Not reported
**Interventions**	Group A: received infiltration of 1.8 mL of 2% lignocaine in addition to 1:100,000 adrenaline and external cold and a vibrating device (Buzzing device as distraction)<br><br>Group B: received infiltration of 1.8 mL of 2% lignocaine in addition to 1:100,000 adrenaline
**Outcomes**	Pain during LA: 1-5 RMS Pictorial Scale and 0-10 the revised Face, Legs, Activity, Cry, Consolability scale

Risk of bias		
**Bias**	**Authors' judgement**	**Support for judgement**
**Random sequence generation**	Low risk	The usage of flip coin method was considered
**Allocation concealment**	Unclear risk	Allocation concealment method was not reported
**Blinding of participants and personnel for all outcomes**	High risk	It was not possible to blind the operator/participants to the intervention
**Blinding of outcome assessors for all outcomes**	Unclear risk	It is unclear whether assessor was blind
**Incomplete outcome data for all outcomes**	Low risk	The authors reported on incomplete data “This study was successfully completed by thirty children with a total of four dropouts”
**Selective outcome reporting**	Low risk	The authors reported all expected outcomes
**Other sources of bias**	Low risk	No other bias

AmruthaVarshini 2021 [61]

Characteristics of included studies	
**Methods**	Study design: RCT, Crossover<br><br>Location: India<br><br>Setting: The Outpatient Department of Paediatric Dentistry
**Participants**	Children requiring local anaesthesia for extraction of maxillary primary molars<br><br>Sample size: 30<br><br>Age: 9-12 years old<br><br>Mean age: Not reported
**Interventions**	Group A: received LA gel application<br><br>Group B: received Ice application<br><br>Group C: received Laser biostimulation with 0.3 W power at a wavelength of 810 nm and probe tip kept 2 mm away from the surface in pulsed mode for 1 minute
**Outcomes**	Pain during LA: 0-10 Wong-Baker FACES scale and 1-4 the Sounds, Eyes, and Motor (SEM) scale

Risk of bias		
**Bias**	**Authors' judgement**	**Support for judgement**
**Random sequence generation**	Low risk	The lottery method was used
**Allocation concealment**	Low risk	Sequentially numbered, opaque, and sealed envelopes were used
**Blinding of participants and personnel for all outcomes**	High risk	It was not possible to blind the operator/participants to the intervention
**Blinding of outcome assessors for all outcomes**	Unclear risk	It is unclear whether assessor was blind
**Incomplete outcome data for all outcomes**	Low risk	The authors evaluated all included participants
**Selective outcome reporting**	Low risk	The authors reported all expected outcomes
**Other sources of bias**	Unclear risk	No baseline characteristics reported

Al-Halabi 2018 [33]

Characteristics of included studies	
**Methods**	Study design: RCT, Parallel groups

	Location: Syria<br><br>Setting: Department of Pediatric Dentistry, Faculty of Dentistry, Damascus University
**Participants**	Children requiring inferior alveolar nerve block<br><br>Sample size: 101<br><br>Age: 6-10 years old<br><br>Mean age: 7.4 years old
**Interventions**	Group A: received IANB with basic behaviour guidance techniques and without distraction aids (n=34)<br><br>Group B: received IANB with audio-visual (AV) eyeglasses 'virtual reality (VR) Box' and wireless headphone (n=33)<br><br>Group C: received IANB with tablet device and wireless headphone (n=34)
**Outcomes**	Pain during LA: 0-5 Wong-Baker FACES scale

**Risk of bias**		
**Bias**	**Authors' judgement**	**Support for judgement**
**Random sequence generation**	Low risk	A randomization website 'Random.org' was used
**Allocation concealment**	Unclear risk	Allocation concealment method was not reported
**Blinding of participants and personnel for all outcomes**	High risk	It was not possible to blind the operator/participants to the intervention
**Blinding of outcome assessors for all outcomes**	High risk	Assessor was not blind
**Incomplete outcome data for all outcomes**	Low risk	The authors reported on incomplete data "one patient was excluded duo to behavioral problems"
**Selective outcome reporting**	Low risk	The authors reported all expected outcomes
**Other sources of bias**	Unclear risk	No baseline characteristics reported

Primosch 2001 [46]

**Characteristics of included studies**	
**Methods**	Study design: RCT, Crossover

	Location: USA<br><br>Setting: Department of Pediatric Dentistry, Faculty of Dentistry, University of Florida
**Participants**	Children requiring bilateral palatal anaesthesia for restoring maxillary molars<br><br>Sample size: 40<br><br>Age: 7-15 years old<br><br>Mean age: 10.75 years old
**Interventions**	Phase 1: received topical anaesthesia benzocaine 20% gel versus Orabase-B (sodium carboxymethylcel- lulose oral adhesive with benzocaine 20%)<br><br>Phase 2: received topical anaesthesia Orabase-B versus EMLA 5% cream (lidocaine 2.5% and prilocaine 2.5% manually mixed in Orabase Plain “(sodium carboxymethylcellulose oral adhesive)
**Outcomes**	Pain during LA: 0-100 Visual Analogue scale

**Risk of bias**		
**Bias**	**Authors' judgement**	**Support for judgement**
**Random sequence generation**	Unclear risk	Participants were randomly allocated
**Allocation concealment**	Unclear risk	Allocation concealment method was not reported
**Blinding of participants and personnel for all outcomes**	High risk	It was not possible to blind the operator to the intervention
**Blinding of outcome assessors for all outcomes**	High risk	Assessor was not blind
**Incomplete outcome data for all outcomes**	Low risk	The authors evaluated all included participants
**Selective outcome reporting**	Low risk	The authors reported all expected outcomes
**Other sources of bias**	Unclear risk	No baseline characteristics reported

Daneshvar 2021 [62]

**Characteristics of included studies**	
**Methods**	Study design: RCT, Crossover<br><br>Location: Iran

	Setting: Department of Pediatric Dentistry, School of Dentistry, Guilan University of Medical Sciences
**Participants**	Children requiring local anaesthesia for pulpotomy of bilateral primary mandibular 2nd molars<br><br>Sample size: 40<br><br>Age: 5-8 years old<br><br>Mean age: 6.72 years old
**Interventions**	Group A: received 4% articaine with epinephrine 1:100,000 by infiltration<br><br>Group B: received 2% lidocaine with epinephrine 1:80,000 by IANB
**Outcomes**	Pain during Pulpotomy: 1-5 the Facial Image Scale

Risk of bias

**Bias**	**Authors' judgement**	**Support for judgement**
**Random sequence generation**	Low risk	Random number table in Excel was used
**Allocation concealment**	Unclear risk	Allocation concealment method was not reported
**Blinding of participants and personnel for all outcomes**	Unclear risk	Insufficient information to make the judgement
**Blinding of outcome assessors for all outcomes**	Unclear risk	Insufficient information to make the judgement
**Incomplete outcome data for all outcomes**	Low risk	The authors evaluated all included participants
**Selective outcome reporting**	Low risk	The authors reported all expected outcomes
**Other sources of bias**	Unclear risk	No baseline characteristics reported

Kamath 2013 [26]

**Characteristics of included studies**	
**Methods**	Study design: RCT, Parallel groups<br><br>Location: India<br><br>Setting: The Narayana Hrudayalaya Dental Clinics
**Participants**	Children requiring local anaesthesia for the dental treatment<br><br>Sample size: 160

	Age: 4-10 years old<br><br>Mean age: 7.6 years old for males and 7.2 years old for females in group A and 7.8 years old for males and 7.6 years old for females in group B
**Interventions**	Group A (Control): used deep breathing (n=80)<br><br>Group B (Intervention): used distraction technique Writing In The Air Using Leg (WITAUL) (n=80)<br><br>LA was given by IANB with Lignocaine 2%
**Outcomes**	Pain during LA: 0-10 the Modified Toddler- Preschooler Post-operative Pain Scale (TPPPS) for children aged 4-5 years old and 0-10 the FACES Pain Scale–Revised (FPS-R) for children aged 6-10 years old

**Risk of bias**		
**Bias**	**Authors' judgement**	**Support for judgement**
**Random sequence generation**	Low risk	The usage of flip coin method was considered
**Allocation concealment**	Unclear risk	Allocation concealment method was not reported
**Blinding of participants and personnel for all outcomes**	High risk	It was not possible to blind the operator/participants to the intervention
**Blinding of outcome assessors for all outcomes**	Unclear risk	It is unclear whether assessor was blind
**Incomplete outcome data for all outcomes**	Low risk	The authors evaluated all included participants
**Selective outcome reporting**	Low risk	The authors reported all expected outcomes
**Other sources of bias**	Unclear risk	No baseline characteristics reported

Asvanund 2015 [51]

**Characteristics of included studies**	
**Methods**	Study design: RCT, Crossover<br><br>Location: Thailand<br><br>Setting: The pediatric dental clinic at the Golden Jubilee Medical Center, Salaya campus, Nakornpathom and dental clinic at Nong Don Community Hospital, Saraburibprovince

**Participants**	Children requiring local anaesthesia for dental treatment of bilateral carious molars<br><br>Sample size: 49<br><br>Age: 5-8 years old<br><br>Mean age: 7 years old
**Interventions**	Group A: received an injection without wearing audio-visual (AV) eyeglasses<br><br>Group B: received an injection with wearing AV eyeglasses<br><br>LA was given by IANB for mandibular teeth and by infiltration for maxillary teeth with 1.5 ml of mepivacaine with 1:100,000 epinephrine
**Outcomes**	Pain during LA: 0-10 the Faces Pain Scale-Revised (FPS-R)

**Risk of bias**		
**Bias**	**Authors' judgement**	**Support for judgement**
**Random sequence generation**	Unclear risk	Participants were randomly allocated
**Allocation concealment**	Unclear risk	Allocation concealment method was not reported
**Blinding of participants and personnel for all outcomes**	High risk	It was not possible to blind the operator/participants to the intervention
**Blinding of outcome assessors for all outcomes**	High risk	Assessors were not blind
**Incomplete outcome data for all outcomes**	Low risk	The authors reported on incomplete data "two who failed to return for a second visit and one refused to wear AV eyeglasses for the second visit"
**Selective outcome reporting**	Low risk	The authors reported all expected outcomes
**Other sources of bias**	Low risk	No other bias

Mittal 2015 [29]

**Characteristics of included studies**	
**Methods**	Study design: RCT, Parallel groups<br><br>Location: India<br><br>Setting: Department of Pedodontics and Preventive Dentistry

**Participants**	Children requiring local anaesthesia for extraction of primary molars<br><br>Sample size: 100<br><br>Age: 8-13 years old<br><br>Mean age: 9.14 years old
**Interventions**	Group A (Control): received buccal and palatal infiltration using traditional syringe (n=50)<br><br>Group B (Intervention): received buccal and palatal infiltration using Wand (n=50)
**Outcomes**	Pain during LA: 0-100 Visual Analogue scale and 1-4 the Sounds, Eyes, and Motor (SEM) scale

**Risk of bias**		
**Bias**	**Authors' judgement**	**Support for judgement**
**Random sequence generation**	Low risk	Random sampling using chit method was considered
**Allocation concealment**	Unclear risk	Allocation concealment method was not reported
**Blinding of participants and personnel for all outcomes**	High risk	It was not possible to blind the operator/participants to the intervention
**Blinding of outcome assessors for all outcomes**	Unclear risk	It is unclear whether assessors were blind
**Incomplete outcome data for all outcomes**	Low risk	The authors evaluated all included participants
**Selective outcome reporting**	Low risk	The authors reported all expected outcomes
**Other sources of bias**	Unclear risk	No baseline characteristics reported

Oztas 2005 [47]

**Characteristics of included studies**	
**Methods**	Study design: RCT, Crossover<br><br>Location: Turkey<br><br>Setting: Not reported
**Participants**	Children requiring local anaesthesia for pulpotomy of contralateral primary mandibular 2nd molars<br><br>Sample size: 25

	Age: 6-10 years old<br><br>Mean age: Not reported
**Interventions**	Group A (Control): received traditional IANB (Inferior alveolar nerve block)<br><br>Group B (Study): received periodontal ligament injection by Wand
**Outcomes**	Pain during preparation, LA and pulpotomy: 0-3 the Eland Color Scale

**Risk of bias**		
**Bias**	**Authors' judgement**	**Support for judgement**
**Random sequence generation**	Unclear risk	Method of randomisation was not reported
**Allocation concealment**	Unclear risk	Allocation concealment method was not reported
**Blinding of participants and personnel for all outcomes**	High risk	It was not possible to blind the operator/participants to the intervention
**Blinding of outcome assessors for all outcomes**	Unclear risk	It is unclear whether assessors were blind
**Incomplete outcome data for all outcomes**	Low risk	The authors evaluated all included participants
**Selective outcome reporting**	Low risk	The authors reported all expected outcomes
**Other sources of bias**	Unclear risk	No baseline characteristics reported

Wambier 2018 [57]

**Characteristics of included studies**	
**Methods**	Study design: RCT, Crossover<br><br>Location: Brazil<br><br>Setting: The School of Dentistry
**Participants**	Children requiring topical anaesthesia for sealant placement on the contralateral permanent mandibular 1st molars under rubber dam<br><br>Sample size: 82<br><br>Age: 8-12 years old<br><br>Mean age: 10.4 years old
**Interventions**	Group A (Control): received placebo gel

	Group B (Study): received the light-cured anaesthetic gel
**Outcomes**	Pain during clamp placement: 0-5 Facial Expression Wong-Baker Scale, 0-10 Numeric Rating Scale (NRS) and 0-10 Face, Legs, Activity, Cry, Consolability (FLACC) Scale

**Risk of bias**		
**Bias**	**Authors' judgement**	**Support for judgement**
**Random sequence generation**	Low risk	A software (www.sealedenvelop.com) was used
**Allocation concealment**	Low risk	Opaque, consecutively numbered and sealed envelopes were used
**Blinding of participants and personnel for all outcomes**	Low risk	The operator and participants were blind to the intervention
**Blinding of outcome assessors for all outcomes**	Unclear risk	It is unclear whether assessors were blind
**Incomplete outcome data for all outcomes**	Low risk	The authors evaluated all included participants
**Selective outcome reporting**	Low risk	The authors reported all expected outcomes
**Other sources of bias**	Low risk	No other bias

Raslan 2021 [42]

**Characteristics of included studies**	
**Methods**	Study design: RCT, Parallel groups<br><br>Location: Syria<br><br>Setting: Department of Paediatric Dentistry, Tishreen University
**Participants**	Children requiring local anaesthesia for extraction of primary molars<br><br>Sample size: 66<br><br>Age: 6-8 years old<br><br>Mean age: 7.37 years old
**Interventions**	Group A: received Paracetamol 160 mg/5 ml 30 mins preoperatively (n=22)<br><br>Group B: received placebo 30 mins preoperatively (n=22)

	Group C: received 100 mg/5 ml ibuprofen 30 mins preoperatively (n=22)<br><br>All analgesics were taken orally
**Outcomes**	Pain after extraction at 3, 4 and 5 hours: 0-10 Wong-Baker FACES scale

**Risk of bias**		
**Bias**	**Authors' judgement**	**Support for judgement**
**Random sequence generation**	Low risk	A randomized table was used
**Allocation concealment**	Low risk	“Group identifiers were included in dark and sealed envelopes with session numbers identical to those assigned to patients by the randomization table”
**Blinding of participants and personnel for all outcomes**	Low risk	The operator and participants were blind to the intervention
**Blinding of outcome assessors for all outcomes**	Low risk	Assessor was blind
**Incomplete outcome data for all outcomes**	Low risk	The authors evaluated all included participants
**Selective outcome reporting**	Low risk	The authors reported all expected outcomes
**Other sources of bias**	Low risk	No other bias

Supplementary file S3: GRADE assessment for the certainty of evidence of the second systematic review

Summary of findings:

Computer Driven LA compared to Conventional LA for children and adolescents having routine dental treatment

Patient or population: children and adolescents having routine dental treatment
Setting: Dental clinic
Intervention: Computer Driven LA
Comparison: Conventional LA

Outcomes	Anticipated absolute effects* (95% CI)		Relative effect (95% CI)	№ of participants (studies)	Certainty of the evidence (GRADE)	Comments
	Risk with Conventional LA	Risk with Computer Driven LA				
Intra-operative Pain	-	SMD **0.03 SD lower** (0.33 lower to 0.27 higher)	-	607 (7 RCTs)	⨁⨁⨁◯ Moderate	See footnotes

***The risk in the intervention group** (and its 95% confidence interval) is based on the assumed risk in the comparison group and the **relative effect** of the intervention (and its 95% CI).

CI: confidence interval; **SMD:** standardised mean difference

GRADE Working Group grades of evidence
High certainty: we are very confident that the true effect lies close to that of the estimate of the effect.
Moderate certainty: we are moderately confident in the effect estimate: the true effect is likely to be close to the estimate of the effect, but there is a possibility that it is substantially different.
Low certainty: our confidence in the effect estimate is limited: the true effect may be substantially different from the estimate of the effect.
Very low certainty: we have very little confidence in the effect estimate: the true effect is likely to be substantially different from the estimate of effect.

Explanations

a. Certainty of the evidence downgraded by 1 level for high risk of bias.

Summary of findings:

Intra-ligament LA compared to Conventional LA for relieving pain in children and adolescents having routine dental treatment

Patient or population: relieving pain in children and adolescents having routine dental treatment
Setting: Dental clinic
Intervention: Intra-ligament LA
Comparison: Conventional LA

Outcomes	Anticipated absolute effects* (95% CI)		Relative effect (95% CI)	№ of participants (studies)	Certainty of the evidence (GRADE)	Comments
	Risk with Conventional LA	Risk with Intra-ligament LA				
Intra-Operative Pain	-	SMD **1.79 SD lower** (2.37 lower to 1.2 lower)	-	111 (2 RCTs)	⊕⊕◯◯ Low[a,b]	

***The risk in the intervention group** (and its 95% confidence interval) is based on the assumed risk in the comparison group and the **relative effect** of the intervention (and its 95% CI).

CI: confidence interval; **SMD:** standardised mean difference

GRADE Working Group grades of evidence
High certainty: we are very confident that the true effect lies close to that of the estimate of the effect.
Moderate certainty: we are moderately confident in the effect estimate: the true effect is likely to be close to the estimate of the effect, but there is a possibility that it is substantially different.
Low certainty: our confidence in the effect estimate is limited: the true effect may be substantially different from the estimate of the effect.
Very low certainty: we have very little confidence in the effect estimate: the true effect is likely to be substantially different from the estimate of effect.

Explanations

a. Certainty of the evidence downgraded by 1 level for high risk of bias.
b. Certainty of the evidence downgraded by 1 level for serious imprecision.

Summary of findings:

Intra-osseous LA compared to Conventional LA for relieving pain in children and adolescents having routine dental treatment

Patient or population: relieving pain in children and adolescents having routine dental treatment
Setting: Dental clinic
Intervention: Intra-osseous LA
Comparison: Conventional LA

Outcomes	Anticipated absolute effects* (95% CI)		Relative effect (95% CI)	№ of participants (studies)	Certainty of the evidence (GRADE)	Comments
	Risk with Conventional LA	Risk with Intra-osseous LA				
Intra-Operative Pain	-	SMD **0.14 SD lower** (0.52 lower to 0.24 higher)	-	188 (1 RCT)	⊕⊕◯◯ Low[a,b]	

***The risk in the intervention group** (and its 95% confidence interval) is based on the assumed risk in the comparison group and the **relative effect** of the intervention (and its 95% CI).

CI: confidence interval; **SMD:** standardised mean difference

GRADE Working Group grades of evidence
High certainty: we are very confident that the true effect lies close to that of the estimate of the effect.
Moderate certainty: we are moderately confident in the effect estimate: the true effect is likely to be close to the estimate of the effect, but there is a possibility that it is substantially different.
Low certainty: our confidence in the effect estimate is limited: the true effect may be substantially different from the estimate of the effect.
Very low certainty: we have very little confidence in the effect estimate: the true effect is likely to be substantially different from the estimate of effect.

Explanations

a. Certainty of the evidence downgraded by 1 level for high risk of bias.
b. Certainty of the evidence downgraded by 1 level for serious imprecision.

Summary of findings:

4% Articaine compared to 2% Lidocaine for LA for relieving pain in children and adolescents having routine dental treatment

Patient or population: LA for relieving pain in children and adolescents having routine dental treatment
Setting: Dental clinic
Intervention: 4% Articaine
Comparison: 2% Lidocaine

Outcomes	**Anticipated absolute effects*** (95% CI)		Relative effect (95% CI)	№ of participants (studies)	Certainty of the evidence (GRADE)	Comments
	**Risk with 2% Lidocaine**	**Risk with 4% Articaine**				
Intra-Operative Pain	-	SMD **1.04 SD lower** (2.18 lower to 0.1 higher)	-	204 (2 RCTs)	⨁⨁◯◯ Low[a,b]	

***The risk in the intervention group** (and its 95% confidence interval) is based on the assumed risk in the comparison group and the **relative effect** of the intervention (and its 95% CI).

CI: confidence interval; **SMD:** standardised mean difference

GRADE Working Group grades of evidence
High certainty: we are very confident that the true effect lies close to that of the estimate of the effect.
Moderate certainty: we are moderately confident in the effect estimate: the true effect is likely to be close to the estimate of the effect, but there is a possibility that it is substantially different.
Low certainty: our confidence in the effect estimate is limited: the true effect may be substantially different from the estimate of the effect.
Very low certainty: we have very little confidence in the effect estimate: the true effect is likely to be substantially different from the estimate of effect.

Explanations

a. Certainty of the evidence downgraded by 1 level for high risk of bias.
b. Certainty of the evidence downgraded by 1 level for serious imprecision.

Summary of findings:

Different methods of topical anaesthesia compared to Conventional topical anaesthesia for LA for relieving pain in children and adolescents having routine dental treatment

Patient or population: LA for relieving pain in children and adolescents having routine dental treatment
Setting: Dental clinic
Intervention: Different methods of topical anaesthesia
Comparison: Conventional topical anaesthesia

Outcomes	**Anticipated absolute effects*** (95% CI)		Relative effect (95% CI)	№ of participants (studies)	Certainty of the evidence (GRADE)	Comments
	**Risk with Conventional topical anaesthesia**	**Risk with Different methods of topical anaesthesia**				
Intra-Operative Pain	-	SMD **0.64 SD lower** (1.38 lower to 0.09 higher)	-	160 (2 RCTs)	⨁⨁◯◯ Low[a,b]	See footnotes

***The risk in the intervention group** (and its 95% confidence interval) is based on the assumed risk in the comparison group and the **relative effect** of the intervention (and its 95% CI).

CI: confidence interval; **SMD:** standardised mean difference

GRADE Working Group grades of evidence
High certainty: we are very confident that the true effect lies close to that of the estimate of the effect.
Moderate certainty: we are moderately confident in the effect estimate: the true effect is likely to be close to the estimate of the effect, but there is a possibility that it is substantially different.
Low certainty: our confidence in the effect estimate is limited: the true effect may be substantially different from the estimate of the effect.
Very low certainty: we have very little confidence in the effect estimate: the true effect is likely to be substantially different from the estimate of effect.

Explanations

a. Certainty of the evidence downgraded by 1 level for high risk of bias.
b. Certainty of the evidence downgraded by 1 level for serious imprecision.

Summary of findings:

Mechanoreceptor and thermal receptor stimulation compared to for relieving pain in children and adolescents having routine dental treatment

Patient or population: for relieving pain in children and adolescents having routine dental treatment
Setting: Dental clinic
Intervention: mechanoreceptor and thermal receptor stimulation

Outcomes	Anticipated absolute effects* (95% CI)		Relative effect (95% CI)	№ of participants (studies)	Certainty of the evidence (GRADE)	Comments
	**Risk with**	**Risk with mechanoreceptor and thermal receptor stimulation**				
Intra-Operative Pain	-	SMD **1.38 SD lower** (2.02 lower to 0.73 lower)	-	930 (10 RCTs)	⨁⨁⨁◯ Moderate[a]	See footnotes

***The risk in the intervention group** (and its 95% confidence interval) is based on the assumed risk in the comparison group and the **relative effect** of the intervention (and its 95% CI).

CI: confidence interval; **SMD:** standardised mean difference

GRADE Working Group grades of evidence
High certainty: we are very confident that the true effect lies close to that of the estimate of the effect.
Moderate certainty: we are moderately confident in the effect estimate: the true effect is likely to be close to the estimate of the effect, but there is a possibility that it is substantially different.
Low certainty: our confidence in the effect estimate is limited: the true effect may be substantially different from the estimate of the effect.
Very low certainty: we have very little confidence in the effect estimate: the true effect is likely to be substantially different from the estimate of effect.

Explanations

a. Certainty of the evidence downgraded by 1 level for high risk of bias.

Summary of findings:

Behavioural Interventions compared to for relieving pain and anxiety in children and adolescents having routine dental treatment

Patient or population: relieving pain and anxiety in children and adolescents having routine dental treatment
Setting: Dental clinic
Intervention: Behavioural Interventions
Comparison:

Outcomes	Anticipated absolute effects* (95% CI)		Relative effect (95% CI)	№ of participants (studies)	Certainty of the evidence (GRADE)	Comments
	**Risk with**	**Risk with Behavioural Interventions**				
Intra-Operative pain	-	SMD **0.5 SD lower** (0.83 lower to 0.18 lower)	-	1130 (13 RCTs)	⊕⊕⊕◯ Moderate[a]	See footnotes
Anxiety	-	SMD **0.17 SD lower** (0.45 lower to 0.11 higher)	-	178 (3 RCTs)	⊕⊕⊕◯ Moderate[b]	See footnotes

***The risk in the intervention group** (and its 95% confidence interval) is based on the assumed risk in the comparison group and the **relative effect** of the intervention (and its 95% CI).

CI: confidence interval; **SMD:** standardised mean difference

GRADE Working Group grades of evidence
High certainty: we are very confident that the true effect lies close to that of the estimate of the effect.
Moderate certainty: we are moderately confident in the effect estimate: the true effect is likely to be close to the estimate of the effect, but there is a possibility that it is substantially different.
Low certainty: our confidence in the effect estimate is limited: the true effect may be substantially different from the estimate of the effect.
Very low certainty: we have very little confidence in the effect estimate: the true effect is likely to be substantially different from the estimate of effect.

Explanations

a. Certainty of the evidence downgraded by 1 level for high risk of bias.
b. Certainty of the evidence downgraded by 1 level for high risk of bias.

Summary of findings:

Pre-emptive oral analgesics compared to Oral placebo solution for relieving pain in children and adolescents having routine dental treatment

Patient or population: relieving pain in children and adolescents having routine dental treatment
Setting: Dental clinic
Intervention: Pre-emptive oral analgesics
Comparison: Oral placebo solution

Outcomes	Anticipated absolute effects* (95% CI)		Relative effect (95% CI)	№ of participants (studies)	Certainty of the evidence (GRADE)	Comments
	**Risk with Oral placebo solution**	**Risk with Pre-emptive oral analgesics**				
Post-Operative Pain	-	SMD **0.77 SD lower** (1.21 lower to 0.33 lower)	-	208 (3 RCTs)	⨁⨁⨁◯ Moderate[a]	

***The risk in the intervention group** (and its 95% confidence interval) is based on the assumed risk in the comparison group and the **relative effect** of the intervention (and its 95% CI).

CI: confidence interval; **SMD:** standardised mean difference

GRADE Working Group grades of evidence
High certainty: we are very confident that the true effect lies close to that of the estimate of the effect.
Moderate certainty: we are moderately confident in the effect estimate: the true effect is likely to be close to the estimate of the effect, but there is a possibility that it is substantially different.
Low certainty: our confidence in the effect estimate is limited: the true effect may be substantially different from the estimate of the effect.
Very low certainty: we have very little confidence in the effect estimate: the true effect is likely to be substantially different from the estimate of effect.

Explanations

a. Certainty of the evidence downgraded by 1 level for high risk of bias..
