# Supplementary material for: Interventions to Reduce Intra-Operative and Post-Operative Pain Associated with Routine Dental Procedures in Children: A Systematic Review and Meta-Analysis
Source: Dent J (Basel). 2024 May 28;12(6):163. doi: 10.3390/dj12060163 (PMC11202832; doi:10.3390/dj12060163)
Supplement: Supplementary file 1 [file dentistry-12-00163-s001.zip › Supplementary file S3.pdf]

Supplementary file S3: GRADE assessment for the certainty of evidence of the second systematic review

Summary of findings:

Computer Driven LA compared to Conventional LA for children and adolescents having routine dental treatment

Patient or population: children and adolescents having routine dental treatment
Setting: Dental clinic
Intervention: Computer Driven LA
Comparison: Conventional LA

Outcomes	Anticipated absolute effects* (95% CI)		Relative effect (95% CI)	№ of participants (studies)	Certainty of the evidence (GRADE)	Comments
	Risk with Conventional LA	Risk with Computer Driven LA				
Intra-operative Pain	-	SMD **0.03 SD lower** (0.33 lower to 0.27 higher)	-	607 (7 RCTs)	⨁⨁⨁◯ Moderate	See footnotes

***The risk in the intervention group** (and its 95% confidence interval) is based on the assumed risk in the comparison group and the **relative effect** of the intervention (and its 95% CI).

CI: confidence interval; **SMD:** standardised mean difference

GRADE Working Group grades of evidence
High certainty: we are very confident that the true effect lies close to that of the estimate of the effect.
Moderate certainty: we are moderately confident in the effect estimate: the true effect is likely to be close to the estimate of the effect, but there is a possibility that it is substantially different.
Low certainty: our confidence in the effect estimate is limited: the true effect may be substantially different from the estimate of the effect.
Very low certainty: we have very little confidence in the effect estimate: the true effect is likely to be substantially different from the estimate of effect.

Explanations

a. Certainty of the evidence downgraded by 1 level for high risk of bias.

Summary of findings:

Intra-ligament LA compared to Conventional LA for relieving pain in children and adolescents having routine dental treatment

Patient or population: relieving pain in children and adolescents having routine dental treatment
Setting: Dental clinic
Intervention: Intra-ligament LA
Comparison: Conventional LA

Outcomes	Anticipated absolute effects* (95% CI)		Relative effect (95% CI)	№ of participants (studies)	Certainty of the evidence (GRADE)	Comments
	Risk with Conventional LA	Risk with Intra-ligament LA				
Intra-Operative Pain	-	SMD **1.79 SD lower** (2.37 lower to 1.2 lower)	-	111 (2 RCTs)	⨁⨁◯◯ Low[a,b]	

***The risk in the intervention group** (and its 95% confidence interval) is based on the assumed risk in the comparison group and the **relative effect** of the intervention (and its 95% CI).

CI: confidence interval; **SMD:** standardised mean difference

GRADE Working Group grades of evidence
High certainty: we are very confident that the true effect lies close to that of the estimate of the effect.
Moderate certainty: we are moderately confident in the effect estimate: the true effect is likely to be close to the estimate of the effect, but there is a possibility that it is substantially different.
Low certainty: our confidence in the effect estimate is limited: the true effect may be substantially different from the estimate of the effect.
Very low certainty: we have very little confidence in the effect estimate: the true effect is likely to be substantially different from the estimate of effect.

Explanations

a. Certainty of the evidence downgraded by 1 level for high risk of bias.
b. Certainty of the evidence downgraded by 1 level for serious imprecision.

Summary of findings:

Intra-osseous LA compared to Conventional LA for relieving pain in children and adolescents having routine dental treatment

Patient or population: relieving pain in children and adolescents having routine dental treatment
Setting: Dental clinic
Intervention: Intra-osseous LA
Comparison: Conventional LA

Outcomes	Anticipated absolute effects* (95% CI)		Relative effect (95% CI)	№ of participants (studies)	Certainty of the evidence (GRADE)	Comments
	Risk with Conventional LA	Risk with Intra-osseous LA				
Intra-Operative Pain	-	SMD **0.14 SD lower** (0.52 lower to 0.24 higher)	-	188 (1 RCT)	⨁⨁◯◯ Low[a,b]	

***The risk in the intervention group** (and its 95% confidence interval) is based on the assumed risk in the comparison group and the **relative effect** of the intervention (and its 95% CI).

CI: confidence interval; **SMD:** standardised mean difference

GRADE Working Group grades of evidence
High certainty: we are very confident that the true effect lies close to that of the estimate of the effect.
Moderate certainty: we are moderately confident in the effect estimate: the true effect is likely to be close to the estimate of the effect, but there is a possibility that it is substantially different.
Low certainty: our confidence in the effect estimate is limited: the true effect may be substantially different from the estimate of the effect.
Very low certainty: we have very little confidence in the effect estimate: the true effect is likely to be substantially different from the estimate of effect.

Explanations

a. Certainty of the evidence downgraded by 1 level for high risk of bias.
b. Certainty of the evidence downgraded by 1 level for serious imprecision.

Summary of findings:

4% Articaine compared to 2% Lidocaine for LA for relieving pain in children and adolescents having routine dental treatment

Patient or population: LA for relieving pain in children and adolescents having routine dental treatment
Setting: Dental clinic
Intervention: 4% Articaine
Comparison: 2% Lidocaine

Outcomes	**Anticipated absolute effects*** (95% CI)		Relative effect (95% CI)	№ of participants (studies)	Certainty of the evidence (GRADE)	Comments
	**Risk with 2% Lidocaine**	**Risk with 4% Articaine**				
Intra-Operative Pain	-	SMD **1.04 SD lower** (2.18 lower to 0.1 higher)	-	204 (2 RCTs)	⨁⨁◯◯ Low[a,b]	

***The risk in the intervention group** (and its 95% confidence interval) is based on the assumed risk in the comparison group and the **relative effect** of the intervention (and its 95% CI).

CI: confidence interval; **SMD:** standardised mean difference

GRADE Working Group grades of evidence
High certainty: we are very confident that the true effect lies close to that of the estimate of the effect.
Moderate certainty: we are moderately confident in the effect estimate: the true effect is likely to be close to the estimate of the effect, but there is a possibility that it is substantially different.
Low certainty: our confidence in the effect estimate is limited: the true effect may be substantially different from the estimate of the effect.
Very low certainty: we have very little confidence in the effect estimate: the true effect is likely to be substantially different from the estimate of effect.

Explanations

a. Certainty of the evidence downgraded by 1 level for high risk of bias.
b. Certainty of the evidence downgraded by 1 level for serious imprecision.

Summary of findings:

Different methods of topical anaesthesia compared to Conventional topical anaesthesia for LA for relieving pain in children and adolescents having routine dental treatment

Patient or population: LA for relieving pain in children and adolescents having routine dental treatment
Setting: Dental clinic
Intervention: Different methods of topical anaesthesia
Comparison: Conventional topical anaesthesia

Outcomes	**Anticipated absolute effects*** (95% CI)		Relative effect (95% CI)	№ of participants (studies)	Certainty of the evidence (GRADE)	Comments
	**Risk with Conventional topical anaesthesia**	**Risk with Different methods of topical anaesthesia**				
Intra-Operative Pain	-	SMD **0.64 SD lower** (1.38 lower to 0.09 higher)	-	160 (2 RCTs)	⨁⨁◯◯ Low[a,b]	See footnotes

***The risk in the intervention group** (and its 95% confidence interval) is based on the assumed risk in the comparison group and the **relative effect** of the intervention (and its 95% CI).

CI: confidence interval; **SMD:** standardised mean difference

GRADE Working Group grades of evidence
High certainty: we are very confident that the true effect lies close to that of the estimate of the effect.
Moderate certainty: we are moderately confident in the effect estimate: the true effect is likely to be close to the estimate of the effect, but there is a possibility that it is substantially different.
Low certainty: our confidence in the effect estimate is limited: the true effect may be substantially different from the estimate of the effect.
Very low certainty: we have very little confidence in the effect estimate: the true effect is likely to be substantially different from the estimate of effect.

Explanations

a. Certainty of the evidence downgraded by 1 level for high risk of bias.
b. Certainty of the evidence downgraded by 1 level for serious imprecision.

Summary of findings:

Mechanoreceptor and thermal receptor stimulation compared to for relieving pain in children and adolescents having routine dental treatment

Patient or population: for relieving pain in children and adolescents having routine dental treatment
Setting: Dental clinic
Intervention: mechanoreceptor and thermal receptor stimulation

Outcomes	Anticipated absolute effects* (95% CI)		Relative effect (95% CI)	№ of participants (studies)	Certainty of the evidence (GRADE)	Comments
	**Risk with**	**Risk with mechanoreceptor and thermal receptor stimulation**				
Intra-Operative Pain	-	SMD **1.38 SD lower** (2.02 lower to 0.73 lower)	-	930 (10 RCTs)	⨁⨁⨁◯ Moderate[a]	See footnotes

***The risk in the intervention group** (and its 95% confidence interval) is based on the assumed risk in the comparison group and the **relative effect** of the intervention (and its 95% CI).

CI: confidence interval; **SMD:** standardised mean difference

GRADE Working Group grades of evidence
High certainty: we are very confident that the true effect lies close to that of the estimate of the effect.
Moderate certainty: we are moderately confident in the effect estimate: the true effect is likely to be close to the estimate of the effect, but there is a possibility that it is substantially different.
Low certainty: our confidence in the effect estimate is limited: the true effect may be substantially different from the estimate of the effect.
Very low certainty: we have very little confidence in the effect estimate: the true effect is likely to be substantially different from the estimate of effect.

Explanations

a. Certainty of the evidence downgraded by 1 level for high risk of bias.

Summary of findings:

Behavioural Interventions compared to for relieving pain and anxiety in children and adolescents having routine dental treatment

Patient or population: relieving pain and anxiety in children and adolescents having routine dental treatment
Setting: Dental clinic
Intervention: Behavioural Interventions
Comparison:

Outcomes	Anticipated absolute effects* (95% CI)		Relative effect (95% CI)	№ of participants (studies)	Certainty of the evidence (GRADE)	Comments
	**Risk with**	**Risk with Behavioural Interventions**				
Intra-Operative pain	-	SMD **0.5 SD lower** (0.83 lower to 0.18 lower)	-	1130 (13 RCTs)	⨁⨁⨁◯ Moderate[a]	See footnotes
Anxiety	-	SMD **0.17 SD lower** (0.45 lower to 0.11 higher)	-	178 (3 RCTs)	⨁⨁⨁◯ Moderate[b]	See footnotes

***The risk in the intervention group** (and its 95% confidence interval) is based on the assumed risk in the comparison group and the **relative effect** of the intervention (and its 95% CI).

CI: confidence interval; **SMD:** standardised mean difference

GRADE Working Group grades of evidence
High certainty: we are very confident that the true effect lies close to that of the estimate of the effect.
Moderate certainty: we are moderately confident in the effect estimate: the true effect is likely to be close to the estimate of the effect, but there is a possibility that it is substantially different.
Low certainty: our confidence in the effect estimate is limited: the true effect may be substantially different from the estimate of the effect.
Very low certainty: we have very little confidence in the effect estimate: the true effect is likely to be substantially different from the estimate of effect.

Explanations

a. Certainty of the evidence downgraded by 1 level for high risk of bias.
b. Certainty of the evidence downgraded by 1 level for high risk of bias.

Summary of findings:

Pre-emptive oral analgesics compared to Oral placebo solution for relieving pain in children and adolescents having routine dental treatment

Patient or population: relieving pain in children and adolescents having routine dental treatment
Setting: Dental clinic
Intervention: Pre-emptive oral analgesics
Comparison: Oral placebo solution

Outcomes	Anticipated absolute effects* (95% CI)		Relative effect (95% CI)	№ of participants (studies)	Certainty of the evidence (GRADE)	Comments
	**Risk with Oral placebo solution**	**Risk with Pre-emptive oral analgesics**				
Post-Operative Pain	-	SMD **0.77 SD lower** (1.21 lower to 0.33 lower)	-	208 (3 RCTs)	⨁⨁⨁◯ Moderate[a]	

***The risk in the intervention group** (and its 95% confidence interval) is based on the assumed risk in the comparison group and the **relative effect** of the intervention (and its 95% CI).

CI: confidence interval; **SMD:** standardised mean difference

GRADE Working Group grades of evidence
High certainty: we are very confident that the true effect lies close to that of the estimate of the effect.
Moderate certainty: we are moderately confident in the effect estimate: the true effect is likely to be close to the estimate of the effect, but there is a possibility that it is substantially different.
Low certainty: our confidence in the effect estimate is limited: the true effect may be substantially different from the estimate of the effect.
Very low certainty: we have very little confidence in the effect estimate: the true effect is likely to be substantially different from the estimate of effect.

Explanations

a. Certainty of the evidence downgraded by 1 level for high risk of bias..
